# Ethnomedicinal uses of the local flora in Chenab riverine area, Punjab province Pakistan

**DOI:** 10.1186/s13002-019-0285-4

**Published:** 2019-02-01

**Authors:** Muhammad Umair, Muhammad Altaf, Rainer W. Bussmann, Arshad Mehmood Abbasi

**Affiliations:** 1 School of Agriculture and Biology and Research Center for Low-Carbon Agriculture, Shanghai Jiao Tong University, Shanghai, 200240 China; 2 Department of Zoology, Women University of Azad Jammu and Kashmir, Bagh, Pakistan; 3 Department of Ethnobotany, Institute of Botany and Bakuriani Alpine Botanical Garden, Ilia State University, Tbilisi, Georgia; 4 Department of Environment Sciences, COMSATS University Islamabad, Abbottabad Campus, Abbottabad, Pakistan

**Keywords:** Ethnobotany, Medicinal plants, Quantitative analysis, Chenab riverine, Pakistan

## Abstract

**Background:**

Because of diverse topographical habitats, the Chenab River wetland harbors a wealth of medicinal and food plant species. This paper presents first quantitative assessment on the ethnobotanical use of plants by the local peoples residing in the Chenab riverine area.

**Methods:**

The ethnobotanical data were collected from six parts of the Chenab River wetland: Mandi Bahuddin, Gujranwala, Gujrat, Sargodha, and Sialkot during 2014 to 2015, using semi-structured interviews. Quantitative indices including informant consensus factor (FCI), relative frequency of citation (RFC), relative importance level (RIL), use value (UV), fidelity level (FL), and corrected fidelity level (CFL) were used to analyze the data.

**Results:**

On the whole, 129 medicinal plant species belonging to 112 genera of 59 families were reported, with herbs as dominant life forms (51%). Poaceae was the leading family with 13 species, and leaves were the most frequently utilized plant parts (28%). Herbal medicines were mostly used in the form of powder or decoction, and were mainly taken orally. *Withania somnifera*, *Solanum surattense*, *Solanum nigrum*, *Azadirachta indica*, *Ficus benghalensis*, *Morus nigra*, *Morus alba*, *Polygonum plebeium*, and *Tribulus terrestris* were among the highly utilized plant species, with highest UV, RFC, RIL, FL, and CFL values. The reported ailments were grouped into 11 categories based on FCI values, whereas highest FIC was recorded for gastrointestinal diseases and glandular diseases (0.41 and 0.34, respectively). The use report (UR) and frequency of citation (FC) depicted strong positive correlation (*r* = 0.973; *p =* 0.01). The value of determination (*r*^2^ = 0.95) indicating 95% variation in UR can be explained in terms of the FC.

**Conclusion:**

The significant traditional knowledge possessed by local communities depicts their strong relation with phytodiversity. Reported data could be helpful in sustainable use and protection of plant species in the Chenab wetland, with special emphasis on medicinal plants. Furthermore, screening of plant-borne active ingredients and in vivo*/*in vitro pharmacological activities could be of interest for novel drug synthesis.

**Electronic supplementary material:**

The online version of this article (10.1186/s13002-019-0285-4) contains supplementary material, which is available to authorized users.

## Background

In traditional health care system, botanical or herbal medicines are based on plant extracts or use of plant parts that may be ingested or applied externally. Herbal drugs are prepared as powders, decoctions, infusions, or as poultice, and are operated in a variety of methods [1]. Herbal medicine is very popular around the globe, with particular reference to South Asia, e.g., Pakistan, India, Bangladesh, and Sri Lanka. The main reasons for the popularity of herbal medicines are (i) the belief that plants are close to nature, hence safer than modern synthetic drugs; (ii) easy accessibility; (iii) plants providing a cheaper method of treatment; and (iv) the idea that plants show less side effects or antagonistic reactions as compared to modern drugs [2]. Still today, the majority of the world population, especially rural people in developing countries like Pakistan, Bangladesh, India, or Nepal, partially or entirely rely on herbal medicine [3].

Ethnobotanical studies are important for the discovery of novel medicines from plant species, which are indigenous heritage of global importance [4]. Medicinal plants help in relieving human distress and are widely used as cosmetics, flavors, oil, bitters, spices, sweeteners, insecticides, and dying agents. About 50 thousands angiospermic plants are used as medicinal purpose [5], out of the total 422 thousands angiospermic plants reported around the globe [6]. An estimated 60% of total population in world, including 80% of the population in underdeveloped countries, use traditional phytomedicine to cure several ailments [7]. In Pakistan, about 2000 plant species have been documented to have biochemical properties. About 600 species are used in different Tibb-e Islami dawakhana (herbal drug markets) by general practitioners (GPs). Besides this, about 50,000 tabibs (GPs of Unani medicine), Ayurveda (GPs of folk medicine), and a number of unlicensed health practitioners spread in remote hilly and rural areas are using more than 200 plant species in herbal drugs [8].

Over the last few decades, there has been a considerable interest worldwide in traditional medicine, specifically in herbal medicines. The World Health Organization (WHO) also described the main role of herbal medicines in preventive, promotive, and curative healthcare system, especially in underdeveloped countries [9]. National Center of Complementary and Alternative medicine (NCCAM), U.S. National Institutes of Health (NIH), classifies complementary and traditional therapies into five major catagories such as whole body system (Unani, Homeopathy, Ayurveda, Chinese medicine); body-mind medicine (mental healing, mediation, prayers); bio-based practices (vitamins, herbs, food); therapeutic and alternative body massages (osteopathy, chiropractic); and bio-field therapies [10]. In Pakistan, herbal drugs have been a strong part of our traditional culture and could have played an important role in providing health care to a large part of the population. In the last few years, mainly three categories, i.e., Ayurveda, Tibb-e-Unani, and homeopathy, are in vogue, whereas Chinese traditional medicine (CTM), aromatherapy, and acupuncture have been introduced in different areas of Pakistan [11].

Chenab River is one of the largest rivers of the Indus basin, spanning a length of 960 km. It is an important wetland of the Punjab, with a flora characteristic of tropical thorn forest [12]. This wetland is rich in biodiversity of vegetables, fodder species, fruits, and medicinal plants. In the Chenab revirine area, the caste system is hundreds of years old and still dominates the social structure of the local communities. For a long time, the people of the Hinjra and Aheer castes have settled in the research area. However, before the partition of Pakistan and India, Bhatti, Kharal, and Tarar were the major castes. Though Muslims always were in the majority, Hindus (Barhaman, Khatri, Kapur, Arorah, Khama, and Chopra), Sikh, and Jatt were also common inhabitants and had great influence on the socio-economic setup. The majority of Hindus and Sikhs migrated to India after partition. Presently, the Chenab riverine area is mainly populated with Muslims, which are divided into Awan, Syyeds, Chattha, Tarar, Kharal, Lodhi, and Hinjrah casts. The majority of the population speaks the Punjabi language, while Siraiki and Urdu are also spoken. Although the young generation is fond of modern culture, the majority of the population prefers Islamic traditions due to strong religious bonds.

The local inhabitants of this area possess significant traditional knowledge and are well aware of plant species used with the aim to treat various diseases. Though, Umair et al. [13], Umair et al. [14], and Mahmood et al. [15] reported ethnobotany of neighboring areas, i.e., Hafizabad, Head Khanki, and Gujranwala districts, but these studies were restricted to these three areas only. The local healers of the Chenab wetland hold knowledge about the utilization of native plant species, particularly to treat health disorders. Therefore, the present study was designed with the aim (i) to compile an inventory of the plant species with medicinal scopes; (ii) to document the traditional knowledge of local communities about medicinal plants along with methods of preparation, dosage, and applications; (iii) to compare the ethnobotanic uses for medicinal scopes with previous reports conducted in neighboring areas; and (iv) to compute importance and fidelity indices of ethnomedicinal uses, which could be helpful to evaluate species or preparations for further evidence-based pharmacological screenings.

## Methods

### The study site

**Fig. 1:**
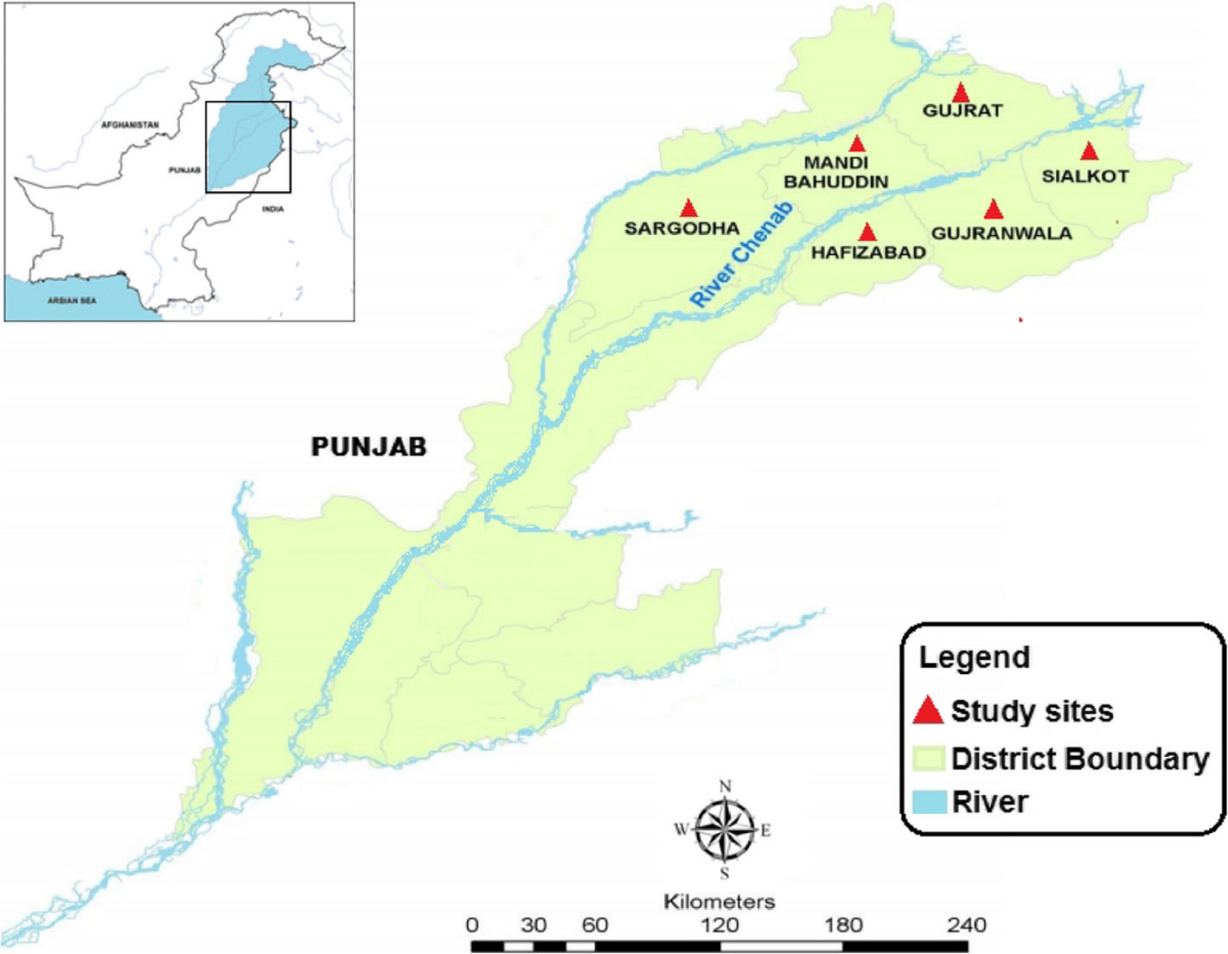
River Chenab and its surrounding areas–Pakistan

The study was conducted on local communities from six districts of Punjab province, Pakistan viz. Hafizabad, Mandi Bahuddin, Gujranwala, Gujrat, Sargodha, and Sialkot sited around the Chenab River (Fig. 1). The source of river Chenab is in Lahul and Spite district in Himachal Pradesh, India. It entered in Pakistan near Diawara town of district Sialkot at 77°–30° E and 32°–50° N (see Additional file 1). The total length of the river is 960 km. The study area spreads over 20,724 km^2^. Climate of this area is semi-arid with an annual average temperature from 48 °C during summer to 1 °C during winter [16]. The mean annual precipitation varies from 340 mm in the south to 780 mm in the upper reaches of Chenab River. The pH of the water is alkaline and averages from 7.9 to 8.1 [17]. The soil is fertile and rich in the medicinal plants diversity due to plain topography. Vegetation of the study area is dominated by grass lands and shrub land [15]. Prominent aquatic vegetation of the study area includes *Hydrilla verticillata*, *Nymphaea lotus*, *Zannichellia palustris*, *Phragmites karka*, *Potamogeton crispus*, *Nelumbo nucifera*, *Typha angustata*, *Vallisneria spiralis*, and Chara species. The natural vegetation of the surrounding plains includes *Tamarix aphylla*, *Prosopis cineraria*, *Saccharum spontaneurn*, *Eleusine compressa*, *Dalbergia sissoo*, and *Ziziphus mauritiana*. Most common weeds of the area are *Tribulus terrestris*, *Xanthium strumarium Euphorbia prostrata*, *Parthenium hysterophorus*, *Achyranthes aspera*, *Cynodon dactylon*, *Amaranthus viridis*, and *Cannabis sativa* [18]. There are about 13 million inhabitants in the study area, with a population density of 594 persons per km^2^. With the growth of human settlement over the centuries, Punjab has cleared most of its forest cover, and over a large part of the Chenab area, bush vegetation has succeeded trees as a result of this land conversion. Nonetheless, a high diversity of grass, herbs, and shrubs persist in this area, which play a key role in herbal medical system [15].

### Documentation and identification of plant species

Field surveys were conducted from April 2014 to July 2015 in four seasons to collect traditional information on therapeutic uses of plant species. Prior consent and approval were taken from departmental ethical committee (Department of Environmental Sciences, COMSATS University Abbottabad Campus) before field survey. Moreover, ethical guidelines of the International Society of Ethnobiology (http://www.ethnobiology.net) were strictly followed during field survey. To collect ethnomedicinal data, questionnaires or semi-structured interviews were conducted with 321 informants (farmers, fishermen, traditional healers/hakeems, housewives, hunters, shopkeepers, and teachers) following the method adopted by Heinrich et al. [19]. Informants were selected on the base of their traditional knowledge on medicinal plants used in health practices. All interviews were conducted after obtaining prior informed consent from the participants (see Additional file 2).

Plant species having medicinal value were collected, dried, pressed, and mounted on herbarium sheets. Voucher specimens were deposited at the Herbarium of Quaid-i-Azam University Islamabad (ISL). Plant species were preliminarily identified during collection, and the identifications were confirmed by expert taxonomist Prof. Dr. Rizwana Aleem Qureshi (Quaid-i-Azam University, Islamabad), and by using the Flora of Punjab and Flora of Pakistan [20–22]. Furthermore, the International Plant Name Index (http://www.ipni.org), the Plant List (www.theplantlist.org), and Germplasm Resources Information Network (GRIN) (http://www.ars-grin.gov/cgi-bin/npgs/html/queries.pl) were used to verify scientific names of plant species, with the nomenclature of families following angiosperm phylogeny group (APG) [23].

### Informant consensus factor

The informant consensus factor (FCI) value is used to describe consensus of informants on the consumption of medicinal plant species and evaluates variability in mode of utilization against reported diseases. All the reported ailments are broadly categorized into 11 categories that include gastrointestinal disorder (GIT), dermatological disorders, glandular disorders, respiratory diseases, sexual diseases, urinary disorders, muscles and skeletal disorders cardiovascular disorders, body energizers, nervous disorders, and ear/nose/eye/mouth diseases (ENEM). FCI values ranges from 0.00 to1.00. High FCI (approaching 1) of an ailment category is recorded when one or few species are reported to be used for that ailment by a large proportion of local people due to their authenticity regarding diseases, whereas a low FCI value indicates that the inhabitants use this species arbitrarily to treat reported ailments. The FCI value is calculated using the formula as described in previous studies [19]:

$$ \mathrm{FCI}=\frac{N_{ur}-{N}_t}{N_{ur}-1} $$

where “*N*_*ur*_” is the total number of use reports for each disease category and “*N*_*t*_” indicates the number of species used in the said category.

### Relative frequency of citation

Relative frequency of citation (RFC) presents the local importance of each species in a study area [24]. To calculate RFC, number of respondents citing a useful species (FC) is divided by total number of respondents in the field survey (*N*) as explained in previous work [25]. RFC value varies from 1 (when all the respondents refer to a plant as a useful one) to 0 (when nobody refers to a plant as a valuable species). RFC was calculated from the following formula:

$$ \mathrm{RFC}=\frac{\mathrm{FC}}{N}\ \left(0<\mathrm{RFC}<1\right) $$

### Relative importance level

The relative importance level (RIL) presents the level of prominence of each species in a study site. The RIL value was calculated using the method described by Friedman et al. [26]. This index is obtained by dividing the number of respondents mentioning a useful species (FC) with total number of respondents of all species (FC*t*). A correction scale (CS) is therefore used, in which all the reported plant species are separated into important and unimportant classes. The relative importance level (RIL) varies from 0 to 1.0, with “1” being full importance of a medicinal plant for particular diseases and “0” no ailment cured by a plant species. When all plant species are frequently used to treat some major ailments, relative importance index would be maximum (1.0); then decrease toward zero as the relative importance of the species diverge away from important side. The RIL index value is logically chosen to equal unity for popular plants (i.e., RIL = 1).

$$ \mathrm{RIL}=\frac{\mathrm{FC}}{{\mathrm{FC}}_t}\ \left(0<\mathrm{RIL}<1\right) $$

### Use value

Use value (UV) is a numerical method that proves the relative importance regarding medicinal uses of plant species and is obtained using the following formula:

$$ {\mathrm{UV}}_i=\frac{\Sigma {U}_i}{n_i} $$

1here UV_*i*_ indicates use value of ith species, *U*_*i*_ is the number of uses recorded for ith species, and *n*_*i*_ shows the number of respondents who mentioned that species.

### Fidelity level

The fidelity level is the percentage of respondents mentioning the uses of a specific plant to treat particular disease. The fidelity level (FL) index was obtained using the given formula [26, 27]:

$$ \mathrm{FL}\ \left(\%\right)=\frac{{\mathrm{FC}}_P}{\mathrm{FC}}\times 100 $$

where FC_*p*_ is the frequency of citation for a particular disease and FC is the total frequency of citation for any particular disease. A high FL index indicates high frequency and popularity of plant utilization for curing a specific disease by the inhabitants of a study site.

### Corrected fidelity level

The corrected fidelity level (CFL) of plant species is used as correction factor to accurately rank the plant species with different FL and RIL values. The CFL is derived from FL, by multiplying FL with RIL values. The CFL index was obtained by the given formula [26, 28].

$$ \mathrm{CFL}=\mathrm{FL}\times \mathrm{RIL} $$

### Pearson correlation coefficient

The Pearson correlation coefficient (PCC) also called as bivariate correlation measures the strength and statistically quantifies the reason of the linear association between two component variables. The data obtained in the interviews were arranged, presented into numeric codes, and subjected to analyses with SPSS 16.0 (SPSS Inc., Chicago, IL). Pearson correlation analysis was analyzed between the frequency of citation (FC) and use reports (UR); the *r*^*2*^ was also measured to calculate species variability and cross relation in term of FC described by variance in UR.

## Results and discussion

### Demographic features of respondents

A total of 321 local informants which is made up of 265 males and 56 females were interviewed. Based on demographic data, these informants were classified into different classes as given in Table 1. In general, traditional healing is a gender-based practice in which both men and women perform this practice [29]. We found a predominance of male participants in survey (82.55%). Such a frequency is likely due to caution of females to converse with male strangers (the interviewers). It was found that among 321 respondents interviewed, 86% were indigenous peoples (IPs) compared to only 14% of traditional health practitioners (THPs). The indigenous peoples were farmers, fishermen, traditional healers/hakeems, housewives, hunters, shopkeepers, and teachers. The age of informants ranged from 18 to 80 years. Maximum informants (23%) were 60 to 80 years old have significant traditional knowledge, whereas little information was provided by young informants. In view of the fact is that traditional knowledge is passed on from one generation to another over time [30]. Approximately, 64 informants (19.94%) were illiterates; other informants had different level of education as follows: < 5 years’ education (18.38%), 8 years’ education (16.82%), 10 years’ education (14.95%), 12 years’ education (11.84%), 14 years’ education (10.28%), and > 16 years’ education (7.79%). This specifies that a certain proportion of people do make a living from using medicinal plants. According to the World Health Organization (WHO), 80% of the world’s people depend on traditional medicine for their primary healthcare needs [9]. THPs have important information on the medicinal uses of plant species to treat different diseases. The maximum numbers of respondents of THPs having more than 20 years’ experience were 14 (Table 1). This may be due to a close relation and wide interaction of indigenous peoples with plant species. Similar distributions were indicated for other areas in Bangladesh [31] and Turkey [32, 33].

**Table 1:** Demographic data of respondents (DDI) from study area

S. #	Variable	Categories	No. of persons	%
1	Gender	Female	56	17.45
		Male	265	82.55
2	Informant category	Traditional health practitioners	45	14.02
		Indigenous peoples	276	85.98
3	Age	≤ 20 years	33	10.28
		20–30 years	42	13.08
		30–40 years	50	15.58
		40–50 years	56	17.45
		50–60 years	65	20.25
		≥ 60 years	75	23.36
4	Educational background	Illiterate	64	19.94
		≤ 5 years	59	18.38
		8 years	54	16.82
		10 years	48	14.95
		12 years	38	11.84
		14 years	33	10.28
		≥ 16 years	25	7.79
5	Experience of THPs	< 2 years	5	11.11
		2–5 years	6	13.33
		5–10 years	12	26.67
		10–20 years	8	17.78
		> 20 years	14	31.11

### Taxonomic classification

Overall, 129 medicinal plant species belonging to 112 genera and 59 families were reported (Table 2). Poaceae was the most dominant family with the largest number of species (13), followed by Asteraceae (12), Fabaceae (11), Moraceae (7), Euphorbiaceae (6), Chenopodiaceae and Malvaceae (5 species each), Amaranthaceae, and Solanaceae (4 species each), whereas other families contributed with only 2 or less species (Table 3). The utilization of plant species belonging to Poaceae was similar in ethnobotanical reports from Pakistan and Bangladesh [34, 35].

**Table 2:** Medicinal plant species used by the local communities of River Chenab and its surrounding areas

S.#	Plant species and accession number	Family	Local name	Common name	Life Habits/ Life forms^a^			Part(s)/mode of utilization^b^	Application mode	Therapeutic uses	Quantitative indices^c^							Previously used^d^
											FC	RFC	UR	UV	RIL	FL	CFL	
1.	*Justicia adhatoda* L. ISNI-RC-86	Acanthaceae	Baykr	Vasak	P	S	W	LE. powder, decoction, juice; FL. decoction; RT. decoction	Oral, Gargle	Malaria, diabetes, asthma, abortion, toothache	43	0.13	28	0.65	0.91	83.7	75	1♦2♦3♦4♦5♦6■7♦8●9♦10■11●12♦13♦14♦15■16♦17♦18■19♦20♦21♦22♦
2.	*Trianthema portulacastrum* L. ISNI-RC-88	Aizoaceae	Itst	Horse parslane	P	H	W	WP. powder; RT. powder, decoction; LE. extract	Oral	Anthelmintic, liver infection, asthma, diuretic, jaundice**,**	27	0.08	11	0.41	0.57	63.0	35	1■2♦3■4♦5♦6■7♦8♦9♦10♦11♦12♦13■14♦15♦16♦17♦18♦19♦20♦21♦22♦
3.	*Achyranthes aspera* L. ISNI-RC-01	Amaranthaceae	Puth kanda	Prickly-Chaff flower	P	H	W	WP. decoction, extract; ST. powder; LE. paste, powder; RT. decoction; RT. juice	Topical, Oral and as Toothbrush	Kidney stone, pneumonia, chest pain, puncture wounds, ulcer, dysmenorrhea, aerodontalgia, asthma	42	0.13	26	0.62	0.89	83.3	73	1♦2■3■4●5♦6■7●8♦9♦10■11♦12♦13♦14●15♦16♦17♦18♦19♦20■21♦22♦
4.	*Alternanthera sessilis* (L.) R.Br. ex DC. ISNI-RC-128	Amaranthaceae	Waglon	Alligator weed	A/P	H	W	LE. juice, cooked, juice; WP. paste; RT. decoction; ST. decoction	Topical, Oral	Eye pain, galactagogue, leucorrhea, snake bite, diarrhea	25	0.08	10	0.40	0.53	56.0	29	1♦2●3●4♦5♦6♦7♦8♦9♦10♦11♦12♦13♦14♦15♦16♦17♦18♦19♦20♦21♦22♦
5.	*Amaranthus spinosus* L. ISNI-RC-02	Amaranthaceae	Gnar	Spiny Pigweed	A	H	W	LE. cooked, juice, extract; RT. juice, decoction; SD. powder; BA. decoction	Gargle, Oral	Vermifuge, dyspepsia, diuretic, odontalgia, cataract, constipation	27	0.08	12	0.44	0.57	59.3	33	1♦2●3♦4■5●6♦7■8●9♦10♦11♦12■13♦14♦15♦16■17♦18♦19♦20♦21♦22♦
6.	*Amaranthus viridis* L. ISNI-RC-03	Amaranthaceae	Ganhar	Slender amaranth	A	H	W	LE. extract, cooked, juice, paste; SD. powder; RT. decoction	Oral and Topical	Painful urination, eye pain, constipation, piles, snakebite, cough and asthma	35	0.11	19	0.54	0.74	68.6	50	1♦2●3♦4●5♦6●7■8♦9♦10■11●12■13■14■15♦16■17♦18●19♦20♦21♦22■
7.	*Mangifera indica* L. ISNI-RC-04	Anacardiaceae	Aamb	Mango	P	T	C	BA. and LE. latex; LE. decoction, paste, infusion; FR. juice; SD. extract	Topical, Oral	Heel fissures, dysentery, febricity, hypoglycemia, blood pressure, snake bite	29	0.09	14	0.48	0.61	62.1	38	1♦2●3♦4♦5♦6♦7♦8♦9■10♦11♦12♦13●14■15♦16♦17♦18♦19♦20♦21♦22■
8.	*Polyalthia longifolia* (Sonn.) Hook.f. & Thomson * ISNI-RC-25	Annonaceae	Ultha ashok	Mast Tree	P	T	C	BA. juice, decoction; LE. Paste	Topical, Oral	Stomachache, body pain, fever, liver tonic	35	0.11	18	0.51	0.74	71.4	52	1♦2♦3♦4♦5♦6♦7♦8♦9♦10♦11♦12♦13♦14♦15♦16♦17♦18♦19♦20♦21♦22♦
9.	*Anethum graveolens* L. ISNI-RC-82	Apiaceae	Sowa	Dil	A/P	H	W/C	SD. powder; LE. infusion, powder	Oral	Gastritis, chronic bronchitis, carminative	39	0.12	22	0.56	0.82	74.4	60	1♦2♦3♦4♦5♦6♦7■8♦9♦10■11♦12♦13■14♦15♦16♦17♦18♦19♦20♦21■22♦
10.	*Nerium oleander* L. ISNI-RC-87	Apocynaceae	Kunair	Oleander	P	S	W	RT. powder; ST.; LE. Juice	Oral, Toothbrush and as Eardrops	Aborficient, toothache, ear infection	41	0.13	25	0.61	0.87	85.4	73	1●2♦3●4●5♦6■7♦8♦9♦10♦11●12♦13♦14♦15■16♦17♦18●19■20■21■22♦
11.	*Pistia stratiotes* L.* ISNI-RC-127	Araceae	Sabs booti	Water lettuce	P	H	W	WP. decoction; LE. juice, extract; RT. paste	Topical, Oral and as Anal	Painful urination, piles, swelling joint, eczema and leprosy, cough and asthma	37	0.12	22	0.59	0.78	73.0	56	1♦2♦3♦4♦5♦6♦7♦8♦9♦10♦11♦12♦13♦14♦15♦16♦17♦18♦19♦20♦21♦22♦
12.	*Schefflera arboricola* (Hayata) Hayata ex Merr. * *ISNI-RC-89*	Araliaceae	Choti chatri	Dwarf schefflera	P	H	C	FR.; RT. extract; RT. paste; WP. decoction	Topical, Oral	Ingestion, blood circulation, cut and wounds, abdominal pain	33	0.10	21	0.64	0.70	69.7	48	1♦2♦3♦4♦5♦6♦7♦8♦9♦10♦11♦12♦13♦14♦15♦16♦17♦18♦19♦20♦21♦22♦
13.	*Calotropis procera* W.T.Aiton ISNI-RC-05	Asclepiadaceae	Akh	Milk weed	P	S	W	LE. extract, paste, poultice. Latex; ST. and LE. decoction; ST. latex	Topical, Oral and as Inhale	Cut and wounds, asthma, odontalgia, hepatitis, T.B., malaria, skin burns, lice-infestation	44	0.14	28	0.64	0.93	86.4	79	1■2■3■4♦5♦6♦7●8♦9♦10■11●12■13■14●15■16♦17●18♦19♦20■21■22■
14.	*Caralluma edulis* Benth. ex Hook.f. ISNI-RC-90	Asclepiadaceae	Chonga	Caralluma	P	H	W	LE. juice, extract; WP. powder	Oral	Anthelmintic, diuretic, diabetes	31	0.10	17	0.55	0.66	67.7	44	1♦2♦3♦4♦5♦6■7♦8♦9♦10■11♦12♦13♦14♦15♦16♦17♦18♦19♦20♦21♦22♦
15.	*Ageratum conyzoides* L. ISNI-RC-06	Asteraceae	Knar	Goat weed	A	H	W	LE. paste, juice, extract; FL. decoction; ST. powder; WP. juice; RT. juice	Topical, Oral and as Eye drop	Jaundice, wounds, febricity, cough, flu, sexual dysfunction,, hair fall, cataract, indigestion	40	0.12	19	0.48	0.85	72.5	60	1♦2■3●4♦5●6♦7■8♦9♦10♦11♦12♦13♦14■15♦16♦17♦18♦19♦20♦21♦22♦
16.	*Artemisia scoparia* Waldst. & Kit. ISNI-RC-91	Asteraceae	Chaou	Wormwood	B	H	W	LE. extract;WP. powder; FL.; SH. Decoction	Topical, Oral	Hair tonic, antidote, malarial fever, laxative	45	0.14	27	0.60	0.95	84.4	79	1♦2♦3♦4♦5♦6■7♦8♦9♦10●11♦12●13♦14♦15■16♦17♦18♦19♦20♦21■22●
17.	*Carthamus oxyacantha* M.Bieb. ISNI-RC-92	Asteraceae	Pholi	Wild safflower	A	H	W	SD. oil; FL.	Oral	Jaundice, obesity, ulcer, male infertility, bronchitis, thrombosis	32	0.10	15	0.47	0.68	71.9	48	1●2♦3♦4♦5♦6●7♦8♦9♦10●11♦12♦13♦14♦15♦16♦17♦18♦19♦20♦21♦22♦
18.	*Cirsium arvense* (L.) Scop. ISNI-RC-07	Asteraceae	Kandaal	Creeping thistle	P	H	W	LE. Juice; FL.; RT. decoction; ST.	Topical, Oral	Ringworm, hepatic ulcer, body tonic, cough, asthma	34	0.11	15	0.44	0.72	73.5	52	1♦2♦3♦4♦5♦6♦7■8♦9♦10♦11♦12♦13♦14♦15♦16♦17●18♦19♦20♦21♦22♦
19.	*Conyza bonariensis* L*.* Cornq. ISNI-RC-08	Asteraceae	Gider booti	Hairy fleabane	A/P	H	W	WP. Extract; RT. decoction; LE. Infusion, juice	Oral	Irregular menstruation, diarrhea, rheumatoid, hyperglycemia, high blood pressure, dysentery	38	0.12	19	0.50	0.80	76.3	60	1♦2♦3♦4♦5♦6♦7■8♦9♦10♦11♦12■13♦14■15♦16♦17♦18♦19■20■21♦22♦
20.	*Lepidium didymum* L. ISNI-RC-09	Asteraceae	Jangli halon	Swine cress	A/B	H	W	ST. powder; LE. infusion; WP. Juice; SH. extract; FL. decoction	Topical, Oral	Bone fracture, tumors, rheumatism, blood purifier, nerve tonic, cold, flu and fever	36	0.11	19	0.53	0.76	77.8	58	1♦2♦3♦4♦5♦6♦7■8♦9♦10♦11♦12♦13♦14♦15♦16♦17♦18♦19♦20♦21♦22♦
21.	*Eclipta prostrata* L. ISNI-RC-10	Asteraceae	Sofed banghara	Trailing eclipta plant	P	H	W	WP. poultice, powder, decoction; LE. juice/tea, powder; RT. decoction	Topical, Oral	Blood purifier, malaria, skin burns, hepatic tumor, hair oil	30	0.09	14	0.47	0.63	70.0	44	1♦2●3●4♦5♦6●7■8♦9♦10♦11♦12■13♦14♦15♦16♦17♦18♦19♦20♦21♦22♦
22.	*Launaea procumbens* Roxb. Ramayya & Rajagopal ISNI-RC-94	Asteraceae	Pili dodhak	Creeping launaea	P	H	W	LE. paste, extract, juice, decoction; WP. decoction	Topical, Oral and as Bath	Sexual disorder, skin infection, febricity, blood purification, renal disorder	25	0.08	9	0.36	0.53	52.0	27	1♦2♦3♦4♦5♦6♦7●8♦9♦10♦11♦12♦13♦14♦15♦16■17♦18♦19♦20♦21♦22♦
23.	*Parthenium hysterophorus* L. ISNI-RC-14	Asteraceae	Gandi boti	Feverfew	A/P	H	W	RT. Juice; FL. powder; WP. decoction, juice; LE. juice, extract	Oral	Laxative, emmenagogue odontalgia, anthelminthic, hyperglycemia, body tonic	29	0.09	12	0.41	0.61	58.6	35	1♦2♦3♦4♦5♦6●7■8♦9♦10■11●12●13■14♦15♦16♦17♦18♦19●20●21♦22♦
24.	*Sonchus asper* Hill. ISNI-RC-11	Asteraceae	Asgandh, Dodak	Spiny leaved Sowhistle	A	H	W	WP. powder; LE. paste; SH. decoction; RT. and L.E. decoction	Topical, Oral	Febricity, cough, bronchial asthma, purgative, wounds, indigestion	27	0.08	12	0.44	0.57	55.6	31	1♦2♦3♦4♦5♦6♦7●8♦9♦10♦11♦12♦13■14■15■16■17♦18●19♦20♦21♦22●
25.	*Taraxacum campylodes* G.E.Haglund ISNI-RC-93	Asteraceae	Peeli booti	Dandilion	A	H	W	LE. paste, powder, decoction; RT. decoction	Topical, Oral	Antidote, diabetes, constipation, liver disorder	28	0.09	11	0.39	0.59	57.1	33	1♦2♦3♦4♦5●6■7♦8●9♦10●11♦12♦13♦14♦15♦16●17♦18■19■20♦21♦22■
26.	*Xanthium strumarium* L. ISNI-RC-13	Asteraceae	Chhota Dhatura	Cocklebur	A	H	W	RT. powder; FR. decoction; LE. powder, decoction	Topical, Oral and as Toothbrush	Malaria, skin ulcer, spinal trauma, indigestion, small pox, scrofulous tumors, odontalgia	26	0.08	11	0.42	0.55	57.7	31	1♦2♦3●4♦5♦6●7■8♦9♦10♦11♦12●13♦14♦15♦16♦17♦18♦19♦20♦21■22■
27.	*Heliotropium strigosum* Willd*.* ISNI-RC-95	Boraginaceae	Gorkh paan	Hairy heliotrope	A/P	H	W	WP. powder, extract; LE. extract	Oral	Blood purifier, urinary tract infection, liver tonic	31	0.10	16	0.52	0.66	71.0	46	1●2♦3♦4♦5♦6■7♦8♦9♦10■11♦12♦13♦14♦15♦16♦17♦18♦19♦20♦21♦22♦
28.	*Trichodesma indicum* (L.) Lehm. ISNI-RC-96	Boraginaceae	Kulfa	Tricodescum	A	H	W	LE. decoction, extract, paste	Topical, Oral	Fever, diarrhea, antidote, rheumatism, diuretic	39	0.12	19	0.49	0.82	71.8	58	1♦2♦3♦4♦5♦6■7♦8♦9♦10♦11♦12♦13♦14♦15■16♦17♦18■19●20●21♦22♦
29.	*Brassica rapa* L. ISNI-RC-16	Brassicaceae	Sarsoon	Field mustard	B	H	C	SD. powder; WP. cocked; LE. decoction	Topical, Oral	Eczema, blood purification, body tonic	33	0.10	16	0.48	0.70	78.8	54	1●2♦3♦4♦5♦6♦7■8♦9♦10♦11♦12♦13●14♦15♦16♦17♦18♦19♦20♦21♦22♦
30.	*Sisymbrium irio* L. ISNI-RC-15	Brassicaceae	Khoob Kalan	London rocket	A	H	W	SD. poultice; FR. powder, decoction, infusion; WP. juice	Topical, Oral	Ophthalmia, indigestion, mumps and measles, skin ulcer, wounds	37	0.12	17	0.46	0.78	78.4	60	1♦2♦3♦4♦5♦6♦7●8♦9♦10●11♦12●13■14♦15♦16♦17♦18♦19♦20♦21■22♦
31.	*Cannabis sativa* L. ISNI-RC-83	Cannabaceae	Bhang	Marijuana	P	S	W/C	LE. paste, extract, infusion; WP. powder; SD. decoction; LE. and SD. juice	Inhale,Topical and as Oral	Constipation, dysentery sedative, snake bite intoxicant, lice infestation, diuretic, purgative, asthma,	46	0.14	29	0.63	0.97	82.6	79	1●2♦3♦4♦5♦6■7●8●9♦10■11♦12■13■14♦15♦16♦17♦18●19♦20♦21■22■
32.	*Capparis decidua* (Forssk.) Edgew ISNI-RC-18	Capparidaceae	kerda, kair	Caper plant	P	T	W	LE. paste; ST. and FL. powder; SH. decoction; BA. powder; SD and FL. decoction; FR.; RT. powder	Topical, Oral	Male sexual dysfunction, hemolytic anemia, anthelminthic, indigestion, hepatic disorder, boils, sciatic and joint pain	35	0.11	17	0.49	0.74	77.1	56	1♦2♦3♦4●5♦6♦7■8♦9■10■11■12♦13♦14■15♦16♦17■18♦19♦20♦21♦22♦
33.	*Stellaria media* (L.) vill. ISNI-RC-19	Caryophyllaceae	Gandhar	Chickweed	A	H	W	LE. paste, poultice, extract WP. decoction; SD.	Topical, Oral	Bone fracture, constipation, itching, wounds, joint pain	30	0.09	14	0.47	0.63	70.0	44	1♦2♦3♦4♦5●6♦7■8♦9♦10♦11♦12♦13♦14♦15♦16■17♦18♦19♦20♦21♦22♦
34.	*Ceratophyllum demersum* L.* ISNI-RC-129	Ceratophyllaceae	Kind-e-Hill	Common contail	P	H	W	LE. juice, decoction, paste	Topical, Oral	Gastric ulcer, diarrhea Biliousness, scorpion stings	40	0.12	27	0.68	0.85	87.5	73	1♦2♦3♦4♦5♦6♦7♦8♦9♦10♦11♦12♦13♦14♦15♦16♦17♦18♦19♦20♦21♦22♦
35.	*Chenopodium album* L. ISNI-RC-20	Chenopodiaceae	Bathu	Lamb’s quarter	A	H	W/C	SH. and FL. juice; WP. cooked; RT. decoction; LE. juice, infusion	Oral	Purgative, indigestion, hepatic disorder, urodynia, rheumatic pain, anthelminthic	47	0.15	31	0.66	0.99	80.9	79	1●2■3♦4♦5♦6■7●8♦9■10■11♦12■13■14■15♦16■17■18♦19♦20♦21■22■
36.	*Chenopodium ambrosioides* L. ISNI-RC-21	Chenopodiaceae	Chandan bathwa	Sweet pigweed	A/P	H	W	SH. and FL. juice; WP. juice; LE. decoction, powder, infusion	Topical, Oral	High blood pressure, irregular menstruation, piles, odontalgia, laxative, indigestion	32	0.10	15	0.47	0.68	71.9	48	1♦2●3♦4♦5●6♦7■8♦9♦10♦11♦12♦13♦14♦15♦16♦17♦18♦19♦20♦21♦22■
37.	*Chenopodium murale* L. ISNI-RC-22	Chenopodiaceae	Karund	Australian-spinach	A	H	W	SD. powder; ST. and LE. paste; WP. decoction; LE. powder, decoction	Topical, Oral and as Snuff	Indigestion, backbone pain, cold and cough, sexual dysfunction, anthelminthic	38	0.12	17	0.45	0.80	76.3	60	1●2♦3♦4♦5♦6♦7■8♦9■10♦11●12■13●14■15♦16♦17♦18♦19♦20♦21♦22♦
38.	*Bassia indica* (Wight) A.J.Scott ISNI-RC-24	Chenopodiaceae	Boi	Indian bassia	A/B	H	W	LE. oil, decoction; FR.	Gargle, Oral	Heart oil, urodynia, odontalgia, tumors	36	0.11	16	0.44	0.76	77.8	58	1♦2♦3♦4♦5♦6♦7●8♦9♦10♦11●12♦13♦14♦15♦16♦17♦18♦19♦20♦21♦22♦
39.	*Suaeda vermiculata* Forssk. ex J.F.Gmel. ISNI-RC-23	Chenopodiaceae	Khaari	Akali seepweed	P	S	W	WP. decoction; ST. ash, decoction; LE. decoction, juice	Topical, Oral	Urodynia, blood purifier, hepatic tumor, snakebite, kidney and bladder stone, hair oil	34	0.11	17	0.50	0.72	79.4	56	1♦2♦3♦4♦5♦6♦7■8♦9♦10●11♦12♦13♦14♦15♦16♦17♦18♦19♦20♦21♦22♦
40.	*Convolvulus arvensis* L. ISNI-RC-25	Convolvulaceae	Lehli/Vahri	Deer’s Foot	A/P	H	W	LE. paste, juice; WP. extract, cooked; RT.	Topical, Oral	Laxative, blood purifier, joint pain, hair oil, ulcer	29	0.09	9	0.31	0.61	65.5	40	1●2♦3♦4♦5♦6●7■8♦9■10●11■12●13●14■15●16♦17●18♦19♦20♦21■22♦
41.	*Bryophyllum pinnatum* (Lam.) Oken ISNI-RC-97	Crassulaceae	Zakhm-i-hayat	Air Plant	P	H	C	LE. extract, paste, juice; RT. infusion	Topical, Oral	Wound healing, dysentery, kidney and pancreatic stone, epilepsy	27	0.08	9	0.33	0.57	63.0	35	1♦2♦3♦4♦5♦6■7♦8♦9♦10■11♦12♦13♦14♦15♦16♦17♦18♦19♦20♦21♦22♦
42.	*Citrullus colocynthis* (L.) Schrad. ISNI-RC-98	Cucurbitaceae	Tuma	Bitter apple	P	H	W	FR.; SD. oil	Topical, Oral	Laxtive, amenorrhea Stomachaches, hair tonic constipation, jaundice	25	0.08	8	0.32	0.53	60.0	31	1■2♦3♦4♦5♦6■7♦8♦9■10■11♦12♦13■14■15♦16♦17■18♦19♦20♦21■22●
43.	*Cucumis melo* L. ISNI-RC-99	Cucurbitaceae	Jangli Kharboza	Pickling melon	A	H	W	FR. decoction; LE. paste; FR.	Topical, Oral	Dysuria, leucorrhea Eczema, purgative	28	0.09	8	0.29	0.59	64.3	38	1♦2♦3♦4♦5♦6♦7♦8♦9♦10■11♦12♦13●14♦15♦16♦17♦18♦19♦20♦21♦22♦
44.	*Cuscuta reflexa* Roxb*.* ISNI-RC-100	Cuscutaceae	Neeli Taar	Giant dodder	A	H	W	SD.; WP. decoction, paste; ST. decoction	Topical, Oral	Urinary disorder, headache, carminative and anodyne, constipation	26	0.08	8	0.31	0.55	61.5	33	1●2■3♦4♦5♦6■7♦8●9♦10●11♦12♦13♦14♦15♦16♦17●18♦19♦20♦21♦22♦
45.	*Cyperus rotundus* L. ISNI-RC-26	Cyperaceae	Daila	Nut grass	P	H	W/C	RH. paste, powder, decoction; LE. decoction, paste; RT. infusion	Topical, Oral	Urodynia, anthelminthic, dermatitis, indigestion, lactation, hypersplenism	47	0.15	32	0.68	0.99	80.9	79	1♦2●3♦4♦5♦6■7●8●9♦10■11♦12♦13♦14♦15♦16♦17♦18♦19♦20♦21♦22♦
46.	*Chrozophora tinctoria* (L.) A.Juss. ISNI-RC-27	Euphorbiaceae	Neeli Booti	Giradol	A	H	W	ST. juice; LE. extract decoction, juice	Eye drop, Oral	Indigestion, Throat ache, vomiting, eye redness	39	0.12	25	0.64	0.82	74.4	60	1♦2♦3♦4♦5♦6♦7■8♦9♦10♦11♦12♦13♦14♦15♦16♦17♦18♦19♦20♦21♦22♦
47.	*Croton bonplandianus* Baill. ISNI-RC-32	Euphorbiaceae	Ban tulsi	Herbel piment	P	H	W	WP. juice, decoction; RT. powder; LE. juice, decoction, poultice; ST. juice	Topical, Oral	Bone Fracture**,** gastric ulcer, hemorrhage, hair tonic, dermatitis, dengue fever, cardiac tonic	31	0.10	21	0.68	0.66	67.7	44	1♦2●3♦4♦5♦6♦7■8♦9♦10♦11♦12♦13♦14♦15♦16♦17♦18♦19♦20♦21♦22♦
48.	*Euphorbia dracunculoides* Lam. ISNI-RC-31	Euphorbiaceae	Bamburi	Dragon spurge	A/P	H	W	FR. juice; LE. powder, paste, juice	Topical, Oral	Lice infestation, head ache, snakebite, skin parasites, epilepsy	35	0.11	23	0.66	0.74	71.4	52	1♦2♦3♦4●5♦6♦7■8♦9♦10♦11♦12♦13♦14♦15♦16♦17♦18♦19♦20♦21♦22♦
49.	*Euphorbia helioscopia* L. ISNI-RC-28	Euphorbiaceae	Chhatri Dodak	Sun euphorbia	A	H	W	WP. powder, latex, juice; SH.; RT.; SD.	Topical, Oral and as Eye drop	Anthelminthic, athlete’s foot, eye sores, asthma, constipation, cholera	39	0.12	23	0.59	0.82	74.4	60	1♦2♦3♦4●5♦6♦7■8♦9■10■11●12■13■14■15♦16♦17♦18♦19■20■21♦22♦
50.	*Euphorbia pilulifera* L. ISNI-RC-29	Euphorbiaceae	Aam dodak, Doddak	Asthma weed	A	H	W	WP. juice, latex, decoction; SD.and FL. powder; LE. juice	Topical, Oral and as Eye drop	Cough, bronchial asthma, indigestion, diarrhea, eye pain, skin burns, cut and wounds	33	0.10	22	0.67	0.70	66.7	46	1♦2♦3♦4♦5♦6♦7■8♦9♦10♦11♦12●13♦14♦15♦16♦17♦18♦19♦20♦21♦22♦
51.	*Euphorbia prostrate* Aiton. ISNI-RC-30	Euphorbiaceae	Doodi Buti	Creeping spurge	P	H	W	LE. infusion, latex, decoction; WP. extract	Topical, Oral	Dysentery, hepatic ulcer, eczema, blood purifier, hyperglycemia, bladder stone, diarrhea	37	0.12	22	0.59	0.78	67.6	52	1♦2●3♦4●5♦6●7■8●9■10■11♦12♦13■14■15♦16♦17♦18■19♦20♦21♦22♦
52.	*Acacia modesta* Wall. ISNI-RC-42	Fabaceae	Phulai	Amritsar gum	P	T	W	ST. extract, gum; ST. and LE. latex; LE. extract; BA. ash, powder	Topical, Oral and as Toothbrush	Aerodontalgia, flatulence, tonic, body tonic, joint pain, bronchitis	32	0.10	21	0.66	0.68	65.6	44	1●2♦3♦4♦5♦6■7■8♦9■10■11■12♦13■14♦15■16♦17●18♦19♦20♦21■22♦
53.	*Acacia nilotica* (L.) Delile ISNI-RC-41	Fabaceae	Kikar	Babul acacia	P	T	W	FL. powder; LE. decoction, paste; BA. powder, ash, decoction; ST. gum;	Oral, Anal and as Toothbrush	Hyperglycemia, indigestion, dysentery, backbone and joints pain, odontalgia, piles, jaundice	45	0.14	31	0.69	0.95	82.2	77	1●2●3♦4♦5♦6■7●8♦9■10■11■12●13■14♦15●16♦17■18■19♦20♦21♦22♦
54.	*Albizia lebbeck* (L.) Benth. ISNI-RC-104	Fabaceae	Sharin	Lebbeck tree	P	T	W	FL.; SD.; ST. (Branches); FR. Decoction	Oral	Sexual disorders, impotency tonic, diuretic, blood purifier, asthma	34	0.11	22	0.65	0.72	67.6	48	1♦2♦3●4♦5♦6■7♦8♦9♦10♦11●12●13♦14♦15♦16♦17■18♦19■20■21♦22♦
55.	*Alhagi maurorum* Medik. ISNI-RC-58	Fabaceae	Jawansa	Camel thorn	P	S	W	BA. decoction, powder; BA. ash; LE. decoction, paste; FL. powder; ST. gum	Oral, Toothbrush and as Anal	Hyperglycemia, indigestion, dysentery, backbone and joints pain, odontalgia, piles, jaundice	38	0.12	25	0.66	0.80	76.3	60	1●2♦3♦4♦5♦6●7●8♦9■10●11♦12♦13♦14■15♦16♦17♦18♦19♦20♦21■22♦
56.	*Cassia fistula* L. ISNI-RC-105	Fabaceae	Amaltas	Golden shower	P	T	W	SD. powder; FL. powder; RT. extract; LE. poultice	Topical, Oral	Gastric, diarrhea, hyperglycemia, pustule	46	0.14	29	0.63	0.97	87.0	83	1♦2●3■4♦5♦6♦7●8●9♦10♦11♦12■13■14♦15●16♦17♦18♦19♦20♦21♦22■
57.	*Dalbergia sissoo* DC. ISNI-RC-57	Fabaceae	Tali	Indian rose wood	P	T	W	WP. decoction; RT. decoction; LE. decoction; FR. powder; RT. infusion; SD. powder	Topical, Oral	Bladder and kidney stone, laxative, piles, bronchial asthma, cough, rheumatism, skin burn, blood purifier	43	0.13	28	0.65	0.91	81.4	73	1●2♦3●4♦5♦6●7●8♦9■10●11●12●13■14■15●16♦17■18♦19■20■21♦22♦
58.	*Indigofera linifolia* (L.f.) Retz. ISNI-RC-107	Fabaceae	Gorakh pan	Common Indigo	A	H	W	WP. decoction; LE. extract; RT. paste; SD.	Topical, Oral	Skin eruption, emollient, swelling joints, tonic	36	0.11	21	0.58	0.76	69.4	52	1♦2♦3♦4♦5♦6●7♦8♦9♦10♦11♦12●13♦14♦15♦16♦17♦18♦19♦20♦21♦22♦
59.	*Melilotus indicus* (L.) All. ISNI-RC-108	Fabaceae	Sinjahi	Sweet clover	A	H	W	LE. paste; WP. powder	Oral	Emollient, diarrhea swellings, bowl complaints, carminative, digestive, skin rash	20	0.06	6	0.30	0.42	40.0	17	1♦2♦3♦4♦5■6♦7♦8♦9■10♦11♦12■13♦14■15♦16■17♦18♦19♦20♦21♦22♦
60.	*Pongamia pinnata* (L.) Pierre ISNI-RC-56	Fabaceae	Such chain	Pongam oiltree	P	T	C	LE. powder; FL. powder; BA. decoction; RT. juice, SD. oil; ST.	Topical, Oral	Tooth pain, rheumatic pain, anthelminthic, flatulence, hyperglycemia, wounds and skin ulcer	30	0.09	12	0.40	0.63	60.0	38	1♦2●3♦4♦5♦6■7■8♦9♦10■11♦12♦13♦14♦15♦16♦17♦18♦19♦20♦21♦22♦
61.	*Prosopis cineraria* (L.) Druce ISNI-RC-43	Fabaceae	Jhand	*Prosopis*	P	S	W/C	FR. powder, paste; ST. decoction; BA. powder; FL. powder; LE. paste, juice	Topical, Oral and as Eye drop	Bladder stone, skin boils, scorpion sting, eye infection, leucorrhoea, dysentery, hepatic ulcer	28	0.09	9	0.32	0.59	57.1	33	1♦2♦3♦4♦5♦6♦7■8♦9■10●11♦12♦13♦14♦15♦16♦17♦18♦19♦20♦21♦22♦
62.	*Prosopis juliflora* (Sw.) DC. ISNI-RC-40	Fabaceae	Mosquet pod	Honey mesquite	P	T	W	BA. powder; LE. poultice, juice; FL. infusion; WP. decoction; ST.	Toothbrush, Topical and as Oral	Bladder stones, tooth pain, breast tumor, bronchial asthma, galactagogue, boils	26	0.08	9	0.35	0.55	53.8	29	1♦2♦3♦4♦5♦6♦7■8♦9♦10♦11♦12♦13♦14♦15♦16♦17♦18♦19♦20♦21■2♦
63.	*Trifolium resupinatum* L. ISNI-RC-55	Fabaceae	Loosin	Reversed clover	A	H	W	FL. powder; WP. infusion, decoction	Gargle, Oral	Throat ache, cough, skin ulcer, sedative, liver tonic, indigestion	22	0.07	9	0.41	0.47	45.5	21	1♦2♦3♦4♦5●6♦7■8♦9♦10♦11♦12♦13♦14♦15♦16♦17♦18♦19♦20♦21♦22♦
64.	*Fumaria indica (Hausskn.)* Pugsley ISNI-RC-101	Fumariaceae	Papra	Indian fumitory	A	H	W	WP. decoction; FL. decoction; FR. Juice; LE. Infusion, tea	Oral	Malaria, constipation, cancer, flu, blood purifier	24	0.07	9	0.38	0.51	50.0	25	1♦2♦3♦4♦5♦6♦7♦8♦9♦10■11●12●13♦14♦15♦16♦17♦18♦19■20■21■22■
65.	*Najas graminea* Delile* ISNI-RC-12	Hydrocharitaceae	Naiad	Ricefield Waternymph	A	H	W	WP.; LE. paste	Topical	Goiter and boils, anticancer	29	0.09	16	0.55	0.61	55.2	33	1♦2♦3♦4♦5♦6♦7♦8♦9♦10♦11♦12♦13♦14♦15♦16♦17♦18♦19♦20♦21♦22♦
66.	*Vallisneria spiralis* L.* ISNI-RC-122	Hydrocharitaceae	Sawala	tape grass	P	H	W	WP.; LE. paste	Topical, Oral	Leucorrhea, rheumatism	27	0.08	16	0.59	0.57	51.9	29	1♦2♦3♦4♦5♦6♦7♦8♦9♦10♦11♦12♦13♦14♦15♦16♦17♦18♦19♦20♦21♦22♦
67.	*Lemna minor* L.* ISNI-RC-17	Lemnaceae	Cheetri	Duck weed	A	H	W	LE. poultice; WP. decoction, powder	Topical, Oral	Skin rashes, antipyretic, diuretic	23	0.07	9	0.39	0.49	47.8	23	1♦2♦3♦4♦5♦6♦7♦8♦9♦10♦11♦12♦13♦14♦15♦16♦17♦18♦19♦20♦21♦22♦
68.	*Abutilon indicum* (L.) Sweet. ISNI-RC-102	Malvaceae	Pelae	Indian mallow	A/B	H	W	LE. paste, decoction; SD.; WP. powder	Topical, Oral	Syphilis, laxative, piles, bronchial asthma	40	0.12	26	0.65	0.85	82.5	69	1♦2■3●4♦5♦6●7♦8♦9♦10●11♦12♦13♦14♦15♦16♦17♦18♦19♦20♦21♦22♦
69.	*Hibiscus rosa-sinensis* L. ISNI-RC-37	Malvaceae	Gurhal	Rose mallow	P	S	C	RT. powder; LE. tea, juice, paste; FL. juice, powder; FL.	Topical, Oral	Sexual dysfunction, leucorrhoea, asthma, skin ulcer, cardiac pain, carminative, diarrhea	41	0.13	25	0.61	0.87	80.5	69	1♦2●3♦4♦5♦6●7■8♦9♦10♦11♦12♦13■14■15♦16♦17♦18♦19♦20♦21♦22♦
70.	*Malva parviflora* L. ISNI-RC-34	Malvaceae	Sonchal	Cheese-weed	A	H	W	SH. and SD. decoction; SH.; LE. decoction, extract, poultice	Topical, Oral	Constipation, abortifacient, sore throat, cough, febricity, scorpion bite	25	0.08	7	0.28	0.53	44.0	23	1●2♦3♦4♦5♦6■7♦8♦9♦10■11●12♦13●14♦15♦16■17♦18♦19♦20♦21♦22♦
71.	*Malvastrum coromandelianum* (L.) Garcke ISNI-RC-35	Malvaceae	Dhamni Buti	False mallow	A	H	W	WP. powder; LE. poultice, decoction, paste	Topical, Oral	Skin sores, eczema, wounds, diarrhea, asthma	40	0.12	23	0.58	0.85	42.5	35	1♦2♦3♦4♦5♦6♦7■8♦9♦10♦11♦12■13●14♦15♦16♦17♦18♦19■20■21♦22♦
72.	*Malvaviscus arboreus* Cav. ISNI-RC-36	Malvaceae	Max mallow	Sleeping hibiscus	P	S	C	LE. juice, decoction; FL. infusion, decoction	Topical, Oral	Throat ache, diarrhea, febricity, Skin eruption	30	0.09	17	0.57	0.63	40.0	25	1♦2♦3♦4♦5♦6♦7■8●9♦10♦11♦12♦13♦14♦15♦16♦17♦18♦19♦20♦21♦22♦
73.	*Marsilea minuta* L.* ISNI-RC-103	Marsiliaceae	Chopatti	Water clover	P	F	W	LE. juice, decoction; FL. infusion, decoction	Topical, Oral	Throat ache, diarrhea, febricity, lice-infestation	38	0.12	21	0.55	0.80	76.3	60	1♦2♦3♦4♦5♦6♦7♦8♦9♦10♦11♦12♦13♦14♦15♦16♦17♦18♦19♦20♦21♦22♦
74.	*Azadirachta indica* A. Juss. ISNI-RC-39	Meliaceae	Neem	Neem	P	T	W/C	LE. decoction, infusion, paste; SD. oil; ST; BA. decoction; LE. paste	Oral, Toothbrush and as Topical	Hyperglycemia, malarial fever, Blood purifier, vermifuge, headache, small pox, aerodontalgia, hepatic ulcer, rheumatic pain	82	0.26	61	0.74	1.00	100.0	100	1■2■3●4♦5♦6♦7●8●9♦10♦11♦12●13■14♦15♦16♦17■18♦19♦20♦21♦22♦
75.	*Melia azedarach* L. ISNI-RC-38	Meliaceae	Dhraikh	Chinaberry	P	T	W/C	ST. decoction; BA. powder; LE. juice, decoction, paste, infusion, extract	Topical, Oral and as Bath	Malaria, itching, wound healing, urinary stones, hypertension, hyperglycemia, blood purification	32	0.10	18	0.56	0.68	75.0	50	1♦2●3♦4♦5♦6●7●8●9♦10■11■12■13■14♦15■16♦17♦18♦19♦20♦21♦22■
76.	*Ficus benghalensis* L. ISNI-RC-106	Moraceae	Bohr	Banyan tree	P	T	W	ST. latex; LE. decoction	Oral	Premature ejaculation, syphilis and gonorrhea, male sexual power	73	0.23	53	0.73	1.00	95.9	96	1■2●3♦4♦5♦6■7♦8♦9♦10●11♦12●13■14♦15♦16♦17●18♦19♦20♦21♦22♦
77.	*Ficus benjamina* L. ISNI-RC-44	Moraceae	Kabar	Weeping Fig	P	T	W	ST. decoction; BA. and LE. cocked; LE. decoction; FR.; WP. powder	Topical, Oral	Stomachache, skin ulcers, flatulence, rheumatic pain, blood purification	36	0.11	19	0.53	0.76	66.7	50	1♦2♦3♦4♦5♦6♦7■8♦9♦10♦11♦12♦13♦14♦15♦16♦17♦18♦19♦20♦21♦22♦15●
78.	*Ficus racemosa* L. ISNI-RC-45	Moraceae	Gular	Cluster tree	P	T	W/C	ST. latex; FR.; BA. decoction, powder; LE. juice	Topical, Oral and as Anal	Diarrhea, adiposity, flatulence, piles, ulcer and boils	34	0.11	16	0.47	0.72	64.7	46	1♦2■3♦4♦5♦6♦7■8♦9♦10♦11♦12♦13♦14♦15♦16♦17♦18♦19♦20♦21♦22♦
79.	*Ficus religiosa* L. ISNI-RC-46	Moraceae	Pipal	Sacred Fig	P	T	W	RT. extract; ST. powder; FR. powder; LE. infusion, paste, decoction	Topical, Oral	Body tonic, bronchial asthma, heart blockage, leucorrhea, ulcer, hypoglycemia	31	0.10	15	0.48	0.66	67.7	44	1♦2●3♦4♦5♦6♦7●8♦9●10♦11♦12■13●14■15♦16♦17●18♦19♦20♦21♦22♦
80.	*Ficus virens* Aiton ISNI-RC-47	Moraceae	Palakh	White Fig	P	T	W	ST. latex; BA. infusion; FR. powder	Oral	Hyperglycemia, ulcer, breast tumor	39	0.12	20	0.51	0.82	74.4	60	1♦2♦3♦4♦5♦6♦7■8♦9♦10♦11♦12♦13♦14♦15♦16♦17♦18♦19♦20♦21♦22♦
81.	*Morus alba* L. ISNI-RC-48	Moraceae	Shahtoot	White mulberry	P	T	C	LE. and BA. decoction; WP. decoction; ST. latex; LE. juice; FR. juice, decoction	Topical, Oral	Cough, constipation, hepatic ulcer, tonsils, snake bite, hypoglycemia	74	0.23	54	0.73	1.00	94.6	95	1♦2♦3♦4♦5♦6♦7■8♦9■10■11♦12■13■14■15■16♦17●18■19♦20♦21■22■
82.	*Morus nigra* L. ISNI-RC-49	Moraceae	Kala toot	Black mulberry	P	T	C	RT. Powder; LE. infusion, decoction; FR. juice, decoction; WP. decoction	Gargle, Oral	Sore throat, cough, asthma, flu, aerodontalgia, hypoglycemia, constipation, vermifuge, carminative	75	0.23	52	0.69	1.00	97.3	97	1♦2♦3♦4♦5♦6●7■8♦9♦10■11●12♦13■14♦15■16♦17♦18■19♦20♦21♦22■
83.	*Eucalyptus camaldulensis* Dehnh. ISNI-RC-51	Myrtaceae	Safaida	River red-gum	P	T	W	LE. oil, extract, juice, decoction	Gargle, Oral	Sinusitis, sore throat, cold, cough, febrifuge, flu	37	0.12	18	0.49	0.78	73.0	56	1■2♦3♦4♦5♦6♦7■8♦9♦10■11♦12♦13●14♦15♦16♦17■18♦19♦20♦21♦22♦
84.	*Psidium guajava* L. ISNI-RC-50	Myrtaceae	Amrud	Guava	P	S	C	FL. decoction; LE. extract, decoction, infusion; FR.	Gargle, Oral	Diarrhea, hyperglycemia, urodynia, carminative, cough, vermifuge, aerodontalgia, febricity, flu	33	0.10	15	0.45	0.70	69.7	48	1♦2♦3♦4♦5♦6♦7■8●9♦10■11♦12♦13■14■15♦16♦17♦18♦19♦20♦21♦22♦
85.	*Nelumbo nucifera* Gaertn.* ISNI-RC-118	Nelumbonaceae	Sacred lotus	*Kanwal*	P	H	W	RT. paste; FL. Juice; LE. paste; RH. paste	Oral, Topical	Piles, diarrhea, headache, ring worm, cardio-tonic	35	0.11	18	0.51	0.74	71.4	52	1♦2♦3♦4♦5♦6♦7♦8♦9♦10♦11♦12♦13♦14♦15♦16♦17♦18♦19♦20♦21♦22♦
86.	*Boerhavia diffusa* L. ISNI-RC-52	Nyctaginaceae	Itsit	Horse-purslane	A/P	H	W	RT. powder, decoction; LE. paste; WP. infusion	Topical, Oral	Dysmenorrhea, cough, snake bite, bronchial asthma, kidney failure, flu	21	0.07	6	0.29	0.44	38.1	17	1●2♦3■4♦5♦6♦7●8♦9♦10♦11♦12♦13♦14♦15♦16♦17♦18♦19♦20♦21♦22♦
87.	*Nymphaea lotus* L.* ISNI-RC-119	Nymphaeaceae	Kamiyan	*Lotus*	P	H	W	LE. and BA. decoction; WP. powder; RT.	Oral	Malarial fever, diuretic, enteritis	29	0.09	13	0.45	0.61	51.7	31	1♦2♦3♦4♦5♦6♦7♦8♦9♦10♦11♦12♦13♦14♦15♦16♦17♦18♦19♦20♦21♦22♦
88.	*Jasminum officinale* L. ISNI-RC-53	Oleaceae	Malti	Poet’s jasmine	P	S	C	LE. extract; FL. decoction; WP. extract; ST. extract, juice	Topical, Oral	Febricity, cough, anthelmintic, scabies, conjunctivitis, diarrhea**,** heart burn	47	0.15	28	0.60	0.99	85.1	83	1♦2♦3♦4♦5♦6♦7■8♦9♦10♦11♦12♦13♦14♦15■16♦17♦18♦19♦20♦21♦22♦
89.	*Jasminum sambac* (L.) Ait. ISNI-RC-54	Oleaceae	Motia	Arabian jasmine	P	S	C	RT. decoction; LE. paste, juice, decoction, extract; FL. juice	Topical, Oral	Conjunctivitis, wound and cuts, emmenagogue, febricity, breast cancer, ulcer, insomnia	45	0.14	26	0.58	0.95	86.7	81	1♦2■3♦4♦5♦6♦7■8♦9♦10♦11♦12♦13♦14♦15♦16♦17♦18♦19♦20♦21♦22♦
90.	*Oxalis corniculata* L*.* ISNI-RC-33	Oxalidaceae	Khatti Buti	Clover sorrel	P	H	W	RT. decoction; WP. powder, decoction; LE. paste, cooked	Topical, Oral and as Eye drop	Diarrhea and dysentery, hepatitis C, wounds, eye inflammation, vermifuge, sexual dysfunction	21	0.07	9	0.43	0.44	42.9	19	1♦2♦3●4♦5●6●7●8●9♦10■11♦12●13■14●15■16●17●18■19♦20♦21♦22●
91.	*Argemone mexicana* L. ISNI-RC-109	Papaveraceae	Stianasi	Mexican poppy	P	H	W	FL. powder; LE. extract	Topical, Oral	Sexual problems, premature ejaculation, spermatoria, emollient, purgative	44	0.14	24	0.55	0.93	86.4	79	1♦2♦3●4♦5●6■7♦8♦9♦10♦11♦12♦13♦14♦15♦16♦17♦18♦19♦20♦21♦22♦
92.	*Avena sativa* L. ISNI-RC-110	Poaceae	Jungli jai	Common oat	A	G	W	WP. powder; LE. infusion	Oral	Nerve tonic, antispasmodic, diuretic	25	0.08	9	0.36	0.53	48.0	25	1♦2♦3♦4♦5♦6■7♦8♦9■10♦11♦12●13■14■15♦16♦17●18♦19♦20♦21♦22♦
93.	*Cenchrus pennisetiformis* Hoschst. & Steud. ISNI-RC-60	Poaceae	Cheetah gha	White buffel grass	A/P	G	W	ST. juice; FR. decoction; LE. infusion, juice, extract	Topical, Oral	Eczema, cough, T.B., asthma, skin irritation, epilepsy, piles	27	0.08	11	0.41	0.57	51.9	29	1♦2♦3♦4♦5♦6♦7■8♦9♦10♦11♦12♦13♦14♦15♦16♦17♦18♦19♦20♦21♦22♦
94.	*Cynodon dactylon* (L.) Pers. ISNI-RC-61	Poaceae	Khanbal gha	Bermuda grass	P	G	W	RT. infusion; WP. juice, paste, decoction; RH. Decoction, oil	Topical, Oral and as Eardrops	Stomachache, bladder stones, eye inflammation, high blood pressure, itching, earache	23	0.07	11	0.48	0.49	47.8	23	1♦2●3♦4♦5♦6●7■8●9■10●11●12■13●14♦15♦16♦17♦18♦19♦20♦21♦22♦
95.	*Dactyloctenium aegyptium* (L.) Willd. ISNI-RC-62	Poaceae	Madhana gha	Crow’s foot grass	A	G	W	WP. paste; RT.; SD.	Topical, Oral	Uterine prolapse, kidney stones**,** indigestion, ulcer and wounds	30	0.09	14	0.47	0.63	46.7	29	1♦2♦3♦4♦5♦6♦7■8♦9♦10♦11♦12♦13♦14♦15♦16♦17♦18♦19♦20♦21♦22♦
96.	*Dichanthium annulatum* (Forssk.) Stapf ISNI-RC-63	Poaceae	Murgha gha	Ringed dichanthium	P	G	W	ST. and LE. decoction; ST. powder; LE. juice, infusion, paste;	Topical, Oral	Abortifacient, diarrhea, indigestion, piles, antispasmodic, scabies	22	0.07	7	0.32	0.47	40.9	19	1♦2♦3♦4♦5♦6♦7■8♦9■10♦11♦12♦13♦14●15♦16♦17♦18♦19♦20♦21♦22♦
97.	*Eleusine indica* (L.) Gaertn. ISNI-RC-64	Poaceae	Madhani	Goose grass	A	G	W	LE. juice; RT. powder; RH. extract; WP. decoction, tea, infusion	Topical, Oral	Febricity, dysentery, irregular menstruation, hyperglycemia, hair tonic, food poisoning	26	0.08	10	0.38	0.55	42.3	23	1♦2♦3♦4♦5♦6♦7■8♦9♦10♦11♦12●13♦14♦15♦16♦17♦18♦19♦20♦21♦22♦15●
98.	*Imperata cylindrica* (L.) Raeusch. ISNI-RC-65	Poaceae	Dabh gha	Cogon grass	P	G	W	RT. decoction; RH. decoction; LE. paste; SH. and LE. paste	Topical, Oral	Body tonic, hypertension, wounds and cuts, urodynia, febricity	24	0.07	9	0.38	0.51	50.0	25	1♦2♦3●4♦5♦6♦7■8●9♦10♦11♦12♦13♦14♦15♦16♦17♦18♦19♦20♦21♦22♦
99.	*Panicum antidotale* Retz. ISNI-RC-123	Poaceae	Sonali	Giant panic	A	G	W	ST. decoction; LE. juice, infusion	Topical, Oral	Respiratory tract infection, appetite, gonorrhea, skin diseases	28	0.09	13	0.46	0.59	46.4	27	1♦2♦3♦4♦5♦6♦7♦8♦9♦10■11♦12♦13♦14♦15♦16♦17♦18♦19♦20♦21♦22♦
100.	*Phragmites karka* (Retz.) Trin. ex Steud. ISNI-RC-120	Poaceae	Nur	*Common reed*	P	G	W	RT. paste; WP. decoction	Topical, Oral	broken bones, rheumatic pain, diaphoretic	41	0.13	25	0.61	0.87	48.8	42	1♦2♦3♦4♦5♦6♦7♦8♦9♦10♦11♦12●13♦14♦15♦16♦17♦18♦19♦20♦21♦22♦
101.	*Saccharum spontaneum* L.* ISNI-RC-124	Poaceae	Kahn	*Wild cane*	P	G	W	RT. decoction; WP. powder; LE. paste	Topical, Oral	Skin eruption, fever, body pain, vermifuge, wounds	35	0.11	19	0.54	0.74	71.4	52	1♦2♦3♦4♦5♦6♦7♦8♦9♦10♦11♦12♦13♦14♦15♦16♦17♦18♦19♦20♦21♦22♦
102.	*Setaria glauca* (L.) P.Beauv. ISNI-RC-66	Poaceae	Bajra	Yellow foxtail	A/P	G	W	SD.; LE. infusion, juice; ST. decoction	Topical	Wound healing, dermatitis, ring worm, tonic, hair tonic	31	0.10	17	0.55	0.66	77.4	50	1♦2♦3♦4♦5♦6♦7■8♦9♦10♦11♦12♦13♦14♦15♦16♦17♦18♦19♦20♦21♦22♦
103.	*Sorghum halepense* (L.) Pers. ISNI-RC-67	Poaceae	Baru	Johnson grass	P	G	W	ST. juice; SD. powder; RT. decoction	Topical, Oral	Stomachache, emollient, boils, cough	33	0.10	19	0.58	0.70	69.7	48	1♦2♦3♦4♦5♦6♦7■8♦9♦10♦11♦12♦13♦14♦15♦16♦17♦18♦19♦20♦21♦22♦
104.	*Triticum aestivum* L. ISNI-RC-59	Poaceae	Kanak	Wheat	A	G	C	SH. decoction; SD. decoction, paste, powder; RT. decoction	Topical, Oral	Colon cancer, wound healing, anemia, asthma, late puberty, hyperglycemia	37	0.12	21	0.57	0.78	59.5	46	1●2♦3♦4♦5♦6♦7●8●9♦10♦11♦12■13♦14♦15♦16♦17♦18♦19♦20♦21♦22♦
105.	*Polygonum plebeium* R. Br. ISNI-RC-68	Polygonaceae	Hind rani	Small knotweed	A	H	W	RT. decoction, LE. extract; SH. decoction; WP. powder, paste	Topical, Oral	Eczema, galactagogue, pneumonia, liver-tonic, heartburn, regular bowl	70	0.22	50	0.71	1.00	91.4	91	1♦2♦3●4♦5♦6●7■8♦9♦10●11♦12♦13♦14♦15♦16♦17♦18♦19♦20♦21♦22♦
106.	*Rumex dentatus* L. ISNI-RC-69	Polygonaceae	Jangli palak	Toothed dock	A	H	W	WP. decoction; LE. and RH. poultice; RT. powder, decoction	Topical, Oral	Eczema, wounds and cuts, constipation, body tonic	35	0.11	21	0.60	0.74	74.3	54	1♦2♦3♦4♦5♦6♦7■8♦9♦10♦11♦12♦13♦14■15■16■17♦18♦19■20■21♦22■
107.	*Eichhornia crassipes* (Mart.) Solms. ISNI-RC-111	Pontederiaceae	Dasi Kulfa	Water-hyacinth	A	H	W	LE. infusion, paste; ST. powder	Topical, Oral	Piles, constipation, cold, flu, respiratory diseases, vermifuge, antiseptic	32	0.10	18	0.56	0.68	68.8	46	1♦2♦3♦4♦5♦6●7♦8♦9♦10♦11♦12♦13♦14♦15♦16♦17♦18♦19♦20♦21♦22♦
108.	*Portulaca quadrifida* L. ISNI-RC-112	Portulacaceae	Kulfa	Common purslane	A	H	W	WP. powder, LE. infusion	Oral	Jaundice, liver and spleen problems	44	0.14	26	0.59	0.93	81.8	75	1♦2♦3●4♦5♦6●7♦8♦9♦10♦11♦12♦13♦14♦15♦16●17♦18♦19♦20♦21♦22♦
109.	*Anagallis arvensis* L. ISNI-RC-70	Primulaceae	Bilibooti	Scarlet pimpernel	A	H	W	ST. powder; LE. and FL. decoction; WP. juice, paste	Topical, Oral	Skin ulcer, leprosy, hepatitis C, epilepsy	36	0.11	20	0.56	0.76	63.9	48	1♦2♦3●4♦5♦6♦7■8♦9●10♦11♦12■13♦14●15♦16♦17♦18♦19♦20♦21♦22♦
110.	*Ranunculus laetus* wall. ex Hook. f. & J.W. Thomson* ISNI-RC-113	Ranunculaceae	Sarsoon booti	Celery-leaved buttercup	A	H	W	LE. paste; FL. extract; SD.; RT. extract	Topical, Oral	Skin infection, conjunctivitis, body tonic, antirheumatic	38	0.12	20	0.53	0.80	73.7	58	1♦2♦3♦4♦5♦6♦7♦8♦9♦10♦11♦12♦13♦14♦15♦16♦17♦18♦19♦20♦21♦22♦
111.	*Ranunculus sceleratus* L*.* ISNI-RC-71	Ranunculaceae	Gul-e-ashrafi	Blister buttercup	A/B	H	W	WP. infusion, juice, decoction; RT. paste; SD.	Topical, Oral	Febricity, body tonic, asthma, muscle hamstring, urinary incontinence, anthelmintic	34	0.11	18	0.53	0.72	58.8	42	1♦2♦3♦4♦5♦6♦7●8♦9♦10♦11♦12♦13♦14♦15♦16♦17♦18♦19♦20♦21♦22♦
112.	*Oligomeris linifolia* (Vahl ex Hornmen) J.F. Macbr.* ISNI-RC-114	Resedaceae	Shootk	Lineleaf oligomeris	A	H	W	SD.; WP. infusion, juice; LE. tea	Oral	Diarrhea, jaundice**,** throat pain and cough, menstrual problems	30	0.09	16	0.53	0.63	53.3	33	1♦2♦3♦4♦5♦6♦7♦8♦9♦10♦11♦12♦13♦14♦15♦16♦17♦18♦19♦20♦21♦22♦
113.	*Ziziphus nummularia* (Burm. f.) Wight and Arn. ISNI-RC-73	Rhamnaceae	baer	Jujube	P	S	W	LE. paste, decoction; BA. decoction; FR. powder	Topical, Oral	Body tonic, hyperglycemia, constipation, scabies, sore throat and cold	28	0.09	13	0.46	0.59	50.0	29	1♦2♦3■4♦5♦6●7■8♦9♦10■11■12♦13♦14♦15♦16♦17♦18■19●20●21■22♦
114.	*Ziziphus mauritiana* Lam. ISNI-RC-72	Rhamnaceae	bairi	Chinese apple	P	T	W	BA. and LE. decoction; BA. powder; LE. decoction, extract, juice; RT. decoction	Topical, Oral, Bath and as Gargle	Chicken pox, ulcers, diarrhea, asthma, toothache, jaundice	40	0.12	24	0.60	0.85	40.0	33	1♦2●3●4♦5♦6♦7■8●9♦10♦11●12●13●14♦15♦16♦17■18♦19♦20♦21●22♦
115.	*Murraya koenigii* (L.) spreng. ISNI-RC-74	Rutaceae	Kari patta	Curry leaf	P	T	C	LE. decoction, juice, infusion, paste; BA. powder; SD.	Topical, Oral	Hyperglycemia, skin eruption, diarrhea, rheumatic pain, eye inflammation, hair oil	38	0.12	21	0.55	0.80	65.8	52	1♦2●3■4♦5♦6♦7■8♦9♦10♦11♦12●13♦14♦15♦16♦17♦18♦19♦20♦21♦22♦
116.	*Salvadora oleoides* Decne*.* ISNI-RC-115	Salvadoraceae	Pelo	Toothbrush tree	P	S	W	ST (Branches); FR.	Oral, Toothbrush	Tonic, stomachache, toothache	34	0.11	16	0.47	0.72	61.8	44	1♦2♦3♦4♦5♦6■7♦8♦9■10■11♦12♦13♦14■15♦16♦17■18♦19♦20♦21♦22♦
117.	*Veronica polita* Fr. ISNI-RC-75	Scrophulariaceae	Veroni	Greyfield speedwell	A	H	W	ST. and LE. cooked; LE. tea, juice; ST. and LE. decoction	Oral	Stomachache, blood purifier, nerve-tonic, cough	42	0.13	4	0.10	0.89	45.2	40	1♦2♦3♦4♦5♦6♦7●8♦9♦10♦11♦12♦13♦14♦15♦16♦17♦18♦19♦20♦21♦22♦
118.	*Misopates orontium* (L.) Raf.* ISNI-RC-116	Scrophulariaceae	Kutta Phool	Snapdragon	A	H	W	WP. extract; LE. poultice, Juice	Topical, Oral and as Eye drop	Contusions, tumors and ulcers, eye inflammation	24	0.07	13	0.54	0.51	45.8	23	1♦2♦3♦4♦5♦6♦7♦8♦9♦10♦11♦12♦13♦14♦15♦16♦17♦18♦19♦20♦21♦22♦
119.	*Datura innoxia* Mill. ISNI-RC-79	Solanaceae	Datura	Thorn apple	P	S	W	WP. powder; SD. paste; LE. decoction, extract; FR.; ST. infusion; RT. decoction	Oral, Inhale and as Topical	Rabies, piles, cough, asthma, lice-infestation, premature ejaculation, purgative, narcotic and sedative	29	0.09	15	0.52	0.61	55.2	33	1♦2♦3●4●5♦6●7■8♦9♦10♦11■12♦13●14♦15♦16♦17♦18♦19♦20♦21♦22♦
120.	*Solanum nigrum* L*.* ISNI-RC-76	Solanaceae	Mako	Night shade	A	H	W	LE. powder, cocked, decoction; LE. extract; LE. and FL. juice; RT. pate; WP. Decoction	Topical, Oral and as Eye drop	Breast cancer, diarrhea, febricity, ulcer, chicken pox, hyperglycemia, piles**,** cardiac pain, sore eyes, cuts and wounds	85	0.26	69	0.81	1.00	100.0	100	1♦2●3●4●5♦6●7●8●9■10■11♦12♦13■14■15■16■17■18■19♦20♦21♦22■
121.	*Solanum surattense* Burm.f. ISNI-RC-77	Solanaceae	Kundiari	Thorny nightshade	P	H	W	WP. cooked, decoction; FR. paste; RT. decoction; LE. and FR. decoction	Oral, Topical	Kidney stones, febricity, heel cracks, anthelmintic, asthma, wound healing, liver tonic, rheumatic arthritis	90	0.28	74	0.82	1.00	94.4	94	1●2●3●4●5♦6●7■8●9■10■11●12♦13♦14■15■16♦17●18●19♦20♦21♦22●
122.	*Withania somnifera* (L.) Dunal. ISNI-RC-78	Solanaceae	Asgandh	Winter cherry	P	H	W	LE. paste, decoction, powder; WP. powder; FR.; FL. powder; RT. powder	Oral, Topical and as Snuff	Malarial fever, stomachache, night mare, hyperglycemia, asthma, irregular menstruation, breast cancer, wounds	95	0.30	80	0.84	1.00	100.0	100	1■2♦3♦4■5♦6■7■8♦9■10■11■12■13■14■15♦16♦17♦18♦19♦20♦21♦22♦
123.	*Pterospermum acerifolium* (L.) Willd ISNI-RC-80	Starculiaceae	Kanakchanpa	Maple-leaved Bayur tree	P	T	W/C	FL. paste, infusion, decoction; BA. powder	Topical, Oral	Piles, vermifuge, impotency, body tonic, swellings	25	0.08	12	0.48	0.53	40.0	21	1♦2♦3♦4♦5♦6♦7■8♦9♦10♦11♦12♦13♦14♦15♦16♦17♦18♦19♦20♦21♦22♦
124.	*Tamarix aphylla* (L.) H.Karst. ISNI-RC-81	Tamaricaceae	Athel tamarisk	Rukh	P	T	W	LE. poultice, paste, decoction; BA. ash	Topical, Oral	Febricity, wound and boils eye infection, cough and cold	34	0.11	17	0.50	0.72	67.6	48	1●2♦3♦4♦5♦6♦7●8♦9■10■11♦12♦13♦14■15♦16♦17♦18♦19♦20♦21♦22♦
125.	*Tamarix dioica* Roxb. ex Roth ISNI-RC-117	Tamaricaceae	Rukh	*Tamarisk*	P	S	W	BA. powder; LE.	Oral	Pile, tonic, cough, diarrhea, antiseptic**,** spleen disorder and liver problems	32	0.10	15	0.47	0.68	68.8	46	1♦2♦3♦4♦5♦6■7♦8♦9♦10♦11♦12♦13♦14♦15♦16♦17■18♦19♦20♦21♦22♦
126.	*Trapa bispinosa* Roxb.* ISNI-RC-126	Trapaceae	Singhara	Water chestnut	A	H	W/C	FR.; SD. powder, paste	Oral	Diarrhea and dysentery, dysuria, body energizer, menstrual disorder	37	0.12	19	0.51	0.78	73.0	56	1♦2♦3♦4♦5♦6♦7♦8♦9♦10♦11♦12♦13♦14♦15♦16♦17♦18♦19♦20♦21♦22♦
127.	*Typha angustata* Bory *&* Chaub*.* ISNI-RC-121	Typhaceae	Kundar	*Long Cattails*	P	H	W	RH. paste; FL.	Oral	Diarrhea and dysentery, mumps and measles, gonorrhea	33	0.10	18	0.55	0.70	69.7	48	1♦2♦3♦4♦5♦6♦7♦8♦9♦10♦11●12♦13♦14♦15♦16♦17♦18♦19♦20♦21♦22♦
128.	*Lantana camara* L. ISNI-RC-84	Verbenaceae	Lantana	Lantana	P	S	W	RT. extract; FL. extract; LE. juice, decoction, paste	Topical, Oral	Ringworm, headache, aerodontalgia, malarial fever, rheumatoid arthritis, cuts and wounds, injuries, cough, cold,	43	0.13	25	0.58	0.91	81.4	73	1♦2♦3♦4♦5●6♦7■8♦9■10♦11♦12■13♦14♦15♦16♦17♦18♦19♦20♦21♦22♦
129.	*Tribulus terrestris* L. ISNI-RC-85	Zygophyllaceae	Gukhro	Puncture vine	A/B	H	W	FR. powder, decoction; LE. paste; WP. powder, decoction	Topical, Oral	Dysentery and diarrhea, urodynia, irregular menstruation, wounds, dyspepsia	61	0.19	41	0.67	1.00	90.2	90	1■2♦3♦4♦5♦6♦7■8♦9■10■11♦12♦13■14♦15●16♦17■18♦19♦20♦21♦22♦

^a^Life habits/life forms: *C* cultivated, *W* wild, *G* grass, *S* shrubs, *H* herbs, *T* trees, *P* perennial, *B* biennial, *A* annual

^b^Plant parts: *RH* rhizome, *BA* bark, *FL* flower, *SD* seed, *WP* whole plant, *SH* shoot, *ST* stem, *RT* root, *FR* fruit, *LE* leaf

^c^Quantitative indices: *FC* frequency of citation, *RFC* relative frequency of citation, *UR* use report, *UV* use value, *RIL* relative importance level, *FL* fidelity level, *CFL* corrected fidelity level

*Plants species which are newly reported in this study

(■) = Plant with similar use(s); (●) = plant with dissimilar use (s); (♦) = plant not reported in previous study

Previously used: (1) Ullah et al. [62]; (2) Mollik et al. [79]; (3) Verma et al. [80]; (4) Rahman et al. [72]; (5) Chaitanya et al. [73]; (6) Mahmood et al. [15]; (7) Umair et al. [13]; (8) Luitel et al. [74]; (9) Ahmed et al. [75]; (10) Malik et al. [76]; (11) Murad et al. [46]; (12) Zahoor et al. [61]; (13) Rehman et al. [77]; (14) Ahmed et al. [78]; (15) Ahmed et al. [81]; (16) Abbasi et al. [82]; (17) Mussarat et al. [83]; (18) Rashid et al. [84]; (19) Amjad et al. [43]; (20) Shaheen et al. [85]; (21) Aziz et al. [86]; (22) Hussain et al. [87]

**Table 3:** Family wise distribution of medicinal plants in the study area

Families	No. of genera	% age contribution	No. of species	% age contribution
Poaceae	13	11.61	13	10.08
Asteraceae	12	10.71	12	9.30
Fabaceae	11	9.82	12	9.30
Moraceae	2	1.79	7	5.43
Euphorbiaceae	3	2.68	6	4.65
Chenopodicaeae	3	2.68	5	3.88
Malvaceae	5	4.46	5	3.88
Amaranthaceae	3	2.68	4	3.10
Solanaceae	3	2.68	4	3.10
Asclepiadaceae	2	1.79	2	1.55
Boraginaceae	2	1.79	2	1.55
Brassicaceae	2	1.79	2	1.55
Cucurbitaceae	2	1.79	2	1.55
Hydrocharitaceae	2	1.79	2	1.55
Meliacea	2	1.79	2	1.55
Myrtaceae	2	1.79	2	1.55
Oleaceae	1	0.89	2	1.55
Polygonaceae	2	1.79	2	1.55
Ranunculaceae	1	0.89	2	1.55
Rhamnaceae	1	0.89	2	1.55
Scharopholariaceae	2	1.79	2	1.55
Tamaricaceae	1	0.89	2	1.55
Acanthaceae	1	0.89	1	0.78
Aizoaceae	1	0.89	1	0.78
Anacardiaceae	1	0.89	1	0.78
Annonaceae	1	0.89	1	0.78
Apiaceae	1	0.89	1	0.78
Apocynaceae	1	0.89	1	0.78
Araceae	1	0.89	1	0.78
Araliaceae	1	0.89	1	0.78
Cannabaceae	1	0.89	1	0.78
Capparidaceae	1	0.89	1	0.78
Caryophyllaceae	1	0.89	1	0.78
Ceratophyllaceae	1	0.89	1	0.78
Convolvulaceae	1	0.89	1	0.78
Crassulaceae	1	0.89	1	0.78
Cuscutaceae	1	0.89	1	0.78
Cyperaceae	1	0.89	1	0.78
Fumariaceae	1	0.89	1	0.78
Lemnaceae	1	0.89	1	0.78
Marsiliaceae	1	0.89	1	0.78
Nelumbonaceae	1	0.89	1	0.78
Nyctaginaceae	1	0.89	1	0.78
Nymphaeaceae	1	0.89	1	0.78
Oxalidaceae	1	0.89	1	0.78
Papaveraceae	1	0.89	1	0.78
Pontederiaceae	1	0.89	1	0.78
Portulacaceae	1	0.89	1	0.78
Primulaceae	1	0.89	1	0.78
Resedaceae	1	0.89	1	0.78
Rutaceae	1	0.89	1	0.78
Salvadoraceae	1	0.89	1	0.78
Starculiaceae	1	0.89	1	0.78
Trapaceae	1	0.89	1	0.78
Typhaceae	1	0.89	1	0.78
Verbenaceae	1	0.89	1	0.78
Zygophyllaceae	1	0.89	1	0.78
Total	112	100	129	100

**Fig. 2:**
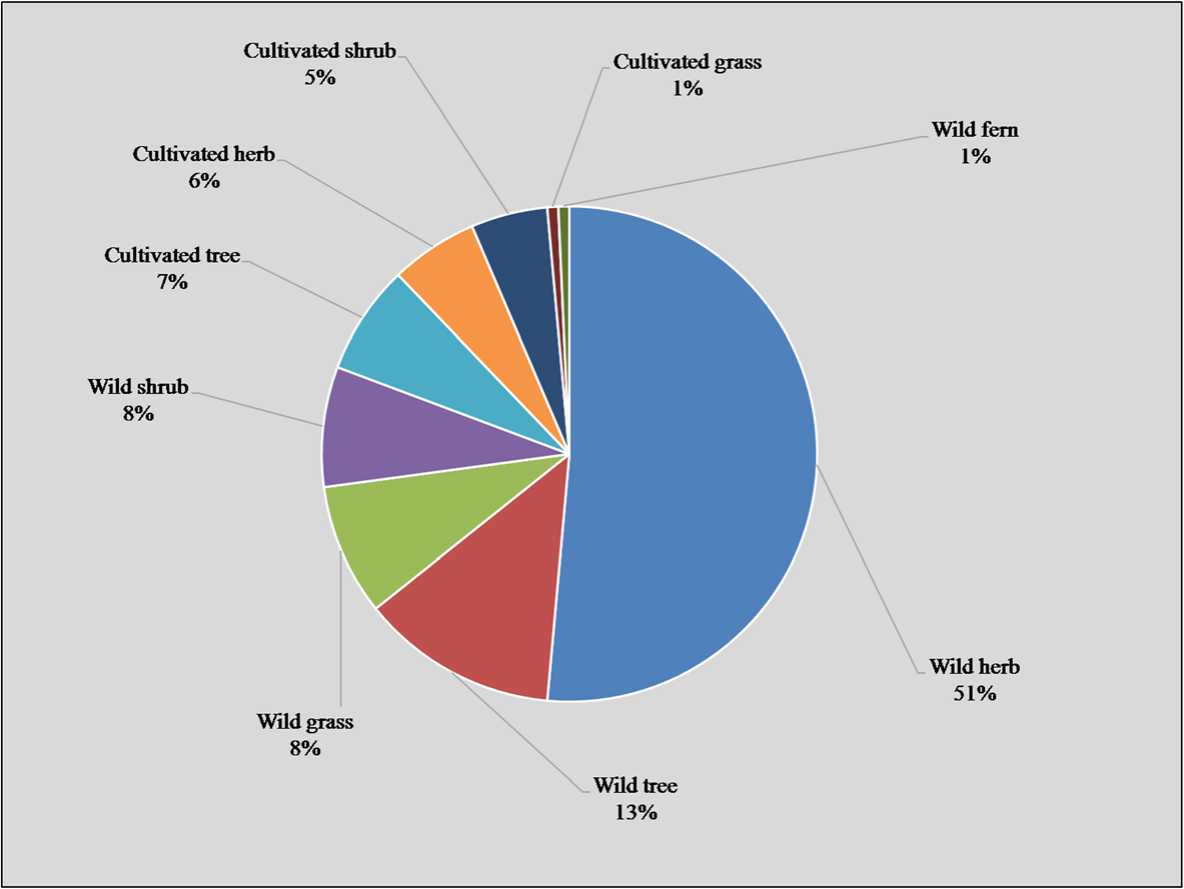
Life forms and habits of medicinal plant species

The wild herbaceous flora constituted 51% of the reported plant species (Fig. 2). Perennial herbs were the most common life habit in the study area. Often, the medicinal plants indicated have perennial life cycles [36, 37]. Wild trees contributed to 13% of the medicinal flora; wild grass and shrubs 8% each; cultivated herbs, shrubs, and grasses 7%, 6%, and 5% respectively; and cultivated grass and wild ferns 1% each (Fig. 2). These findings were similar to previous reports [1, 35]. The common use of wild herbs may be due to their easy availability and efficiency in the treatment of different ailments compared to other life habit. The Engineers India Research Institute (EIRI) [38] reported that wild herbs are more efficient and effective for use in medicines than those grown in garden. Probably, traditional healers used mostly herbs and trees compared to other life forms as medicine due to their availability in nature [39]. Local people usually collected medicinal plants from roadsides, swamp or swamp edges, woodlots, wet grasslands, grassland, bush land, forest, forest edge, fallow land, home garden, and cropland. Species range limits are alienated by the species ecological niche [40], which are often found to be linked with spatial gradients in ecological factors (e.g., precipitation, temperature) and are explained by a set of factors, e.g., climate, habitat structure, and predators or competitors pairs [41]. According to the local informants, herb sellers often collect plants from the wild and supply to herbal market (Pansara) without paying any attention to their conservation. Although some of the listed plants are presented in the study area, some of them are rare due to harvesting or deforestation.

### Plant part(s) used

**Fig. 3:**
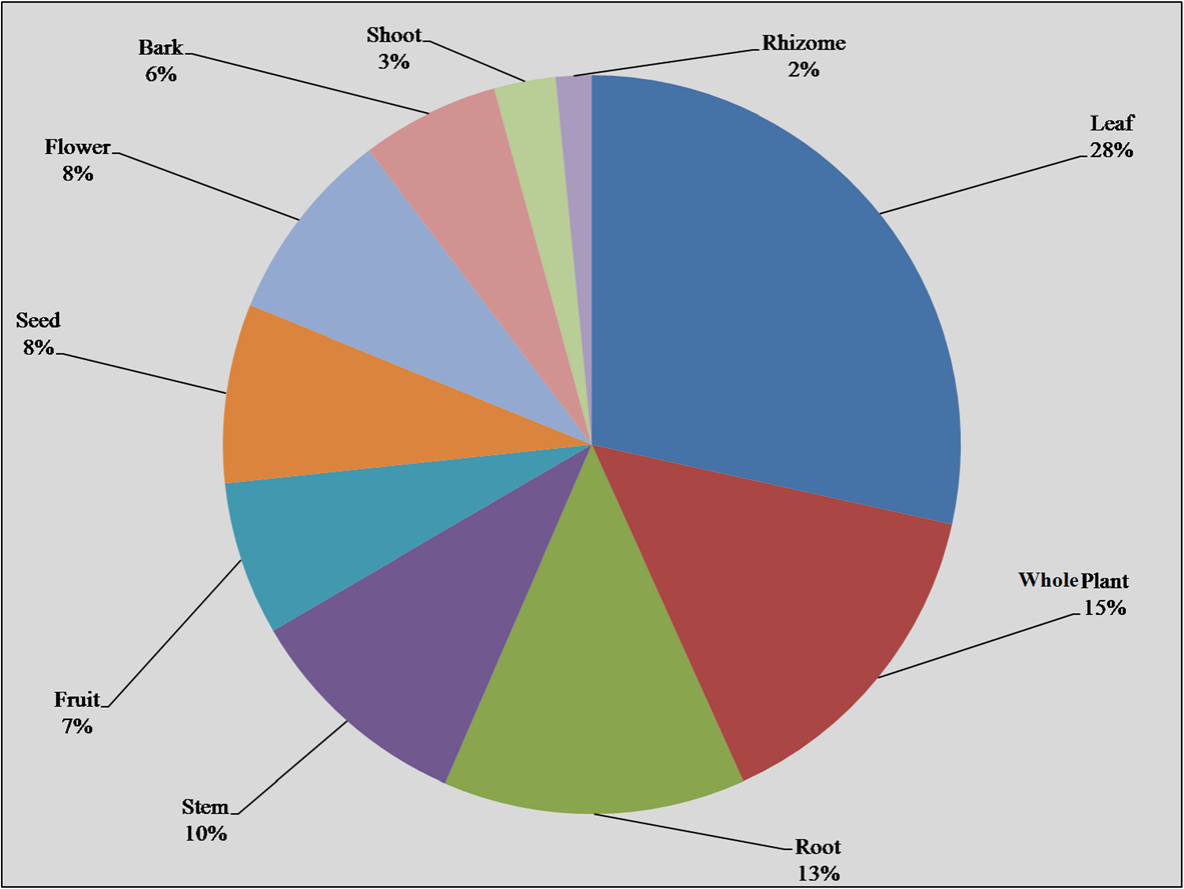
Plant parts used in traditional recipes

The use of plant parts in the preparation of recipes depends upon their availability and knowledge of local people. Leaves were the most frequently utilized plant part with 28% applications in traditional herbal medicine, followed by whole plant (15%), root (13%), stem (10%), seed and flower (8% each), fruit (7%), bark (6%), shoot (3%), and rhizome (2%) (Fig. 3). Leaves are commonly used in herbal medicines because they are rich in bioactive secondary metabolites. Leaves are the main photosynthetic organs and also act as storages for exudates or photosynthates; some of which defend the plants against destructive entities or are of medicinal values to the human body [24, 42]. In previous studies, leaves were also reported as the most frequently utilized plant part [13, 43]. Apart from leaves, the use of whole plants has also been reported in many studies [44–46]. In some cases, the same plant part was used to treat different ailments, e.g., leaves of *Withania somnifera* were taken orally to treat asthma and malarial disease, and applied externally to heal wounds. Similar uses of plants parts of many other species are mentioned in Table 2.

### Toxic plants

Some plant species such as *Croton sparsiflorus*, *Datura innoxia*, *Lantana camara*, *Nerium oleander*, *Calotropis procera*, *Solanum* spp., *Euphobia* spp*.*, and *Ranunculus sceleratus* show toxic effects, if taken in excessive amount [13, 47]. *Nerium oleander* (Kunair) causes gastrointestinal disorder (laxative effect) and mental instability (hemorrhage) when used in excess. Likewise, *Lantana camara* (Lantana) is claimed to cause itchy feelings. The approach for drug development from plant species depends on several ways in which this can be done, including toxicity, chemical content, traditional use, randomized selection, or combination of several criteria. Beneficial or adverse effects of plant-based medicines depend on method of herbal drug preparation and its utilization in herbal medicine [48]. In general, the indigenous peoples of the study area use above-mentioned species in minimal quantities to avoid their poisonous effects, which suggest that they may have at least some empiric knowledge of their dangerousness.

### Mode of preparation and application

**Fig. 4:**
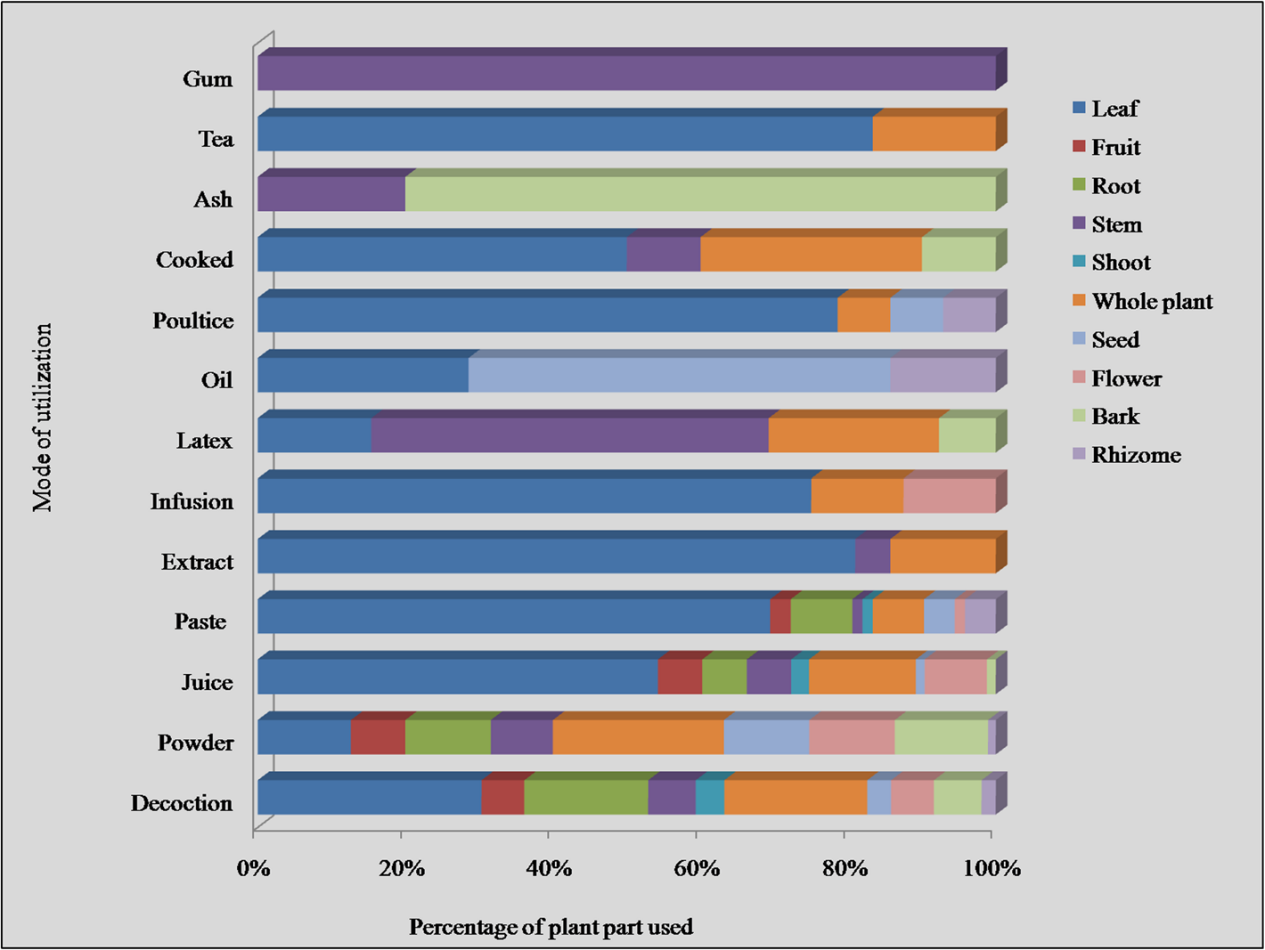
Preparations used in herbal recipes

Herbal medications were prescribed in different forms including powder, decoction, juice, extract, paste, poultice, infusion, ash, etc. (Fig. 4). Decoction was the most commonly used method of herbal preparation with 31%, followed by powder, juice, paste, and extract (19, 17, 14, and 4%, respectively), while the remaining preparations (infusion, poultice, latex, cooked food, oil, tea, ash, and gum) were used for less than 3% of indications. According to Umair et al. [13], decoction was the most used method for herbal preparations in Hafizabad region of Punjab province. Decoctions are often used as one of the major forms of preparations in traditional healthcare system, because they are easy to prepare by mixing herbs with water, tea, or soup [49, 50]. To make decoctions, plant parts are boiled in water until the original volume of the water is reduced to one-fourth [51], whereas plant extract is prepared by crushing or squeezing the plant parts before extraction [52].

Usually, traditional recipes were based on a single plant species. However, in some cases, more than one plant species was used in drug preparation [53]. For instance, the treatment of cough and asthma was done by using a decoction prepared from *S*. *surattense* and *Tinospora cordifolia*. Yamamoto et al. [54] reported that a traditional herbal medicine prepared from eight medicinal plants (Dai-Saiko-to) is used to lower the lipid levels in human body suffering from diabetic hyperlipidemia. In most herbal preparations, water was used as a solvent; however, honey, oil, milk, or tea were also used to enhance the acceptability and hypothesizing their implication in the enhancement of the medicinal properties of the preparation, e.g., root powder of *Boerhavia diffusa* is commonly mixed with honey and used to treat cough, asthma, and flu.

**Fig. 5:**
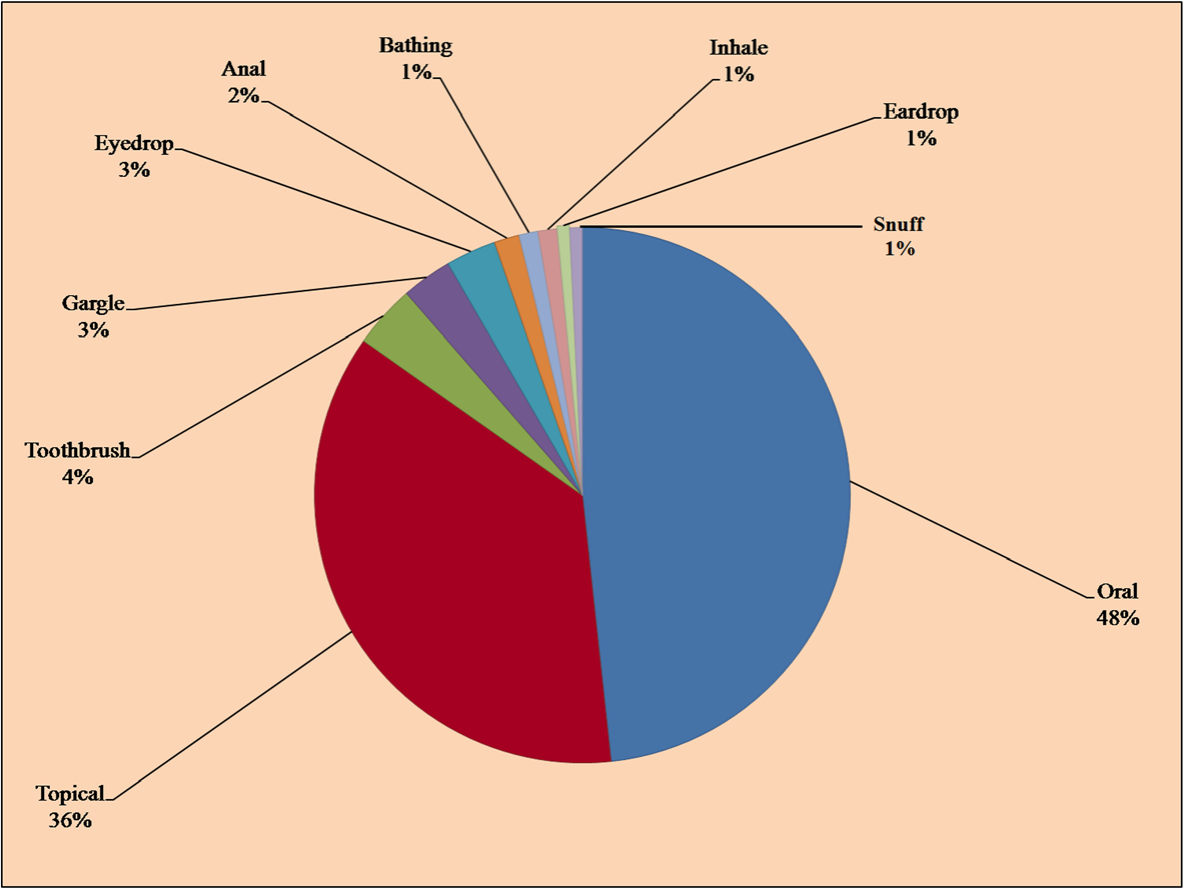
Mode of application of medicinal plants

In the present work, plant-based medications were most frequently utilized to treat different ailments including gastrointestinal disorders (stomachache, gastric ulcer, gas trouble, intestinal worms, vomiting, constipation, dysentery, diarrhea), respiratory problems (asthma, cough, flu, throat ache), skin infections (chicken pox, measles, eczema, rashes, cuts, and wounds), fever, diabetes, kidney problems, cancer, toothache, earache, eye pain, cardiac problems, jaundice, inflammation, menstrual disorders, piles, bone fracture, rheumatism, snake bite, scorpion sting, milk production, and general weakness. The most often utilized mode of administration was oral (48%), followed by topical (36%), as toothbrush (4%), eye drops and gargle (3% each), anal application (2%) and bathe, inhale, eardrops, and snuff (1% each) (Fig. 5). Similar modes of applications were reported in Hafizabad district [13].

It has been reported that oral mode of administration is the most preferred route (76%) among the communities of Gujranwala district, Pakistan [15]. The practice of oral administration may be linked to the use of some additives or solvents (milk, tea, hot coffee, fruit juice, and water) that are commonly believed to serve as a vehicle to transport the herbal medicines. The additives or solvents are also important to improve the taste, minimize soreness, and decrease adverse effects such as diarrhea, vomiting, and increase the efficacy and healing conditions [55]. These results are in agreement to other studies [31, 56]. Leaves of *Melia azedarach* and *Zizyphus mauritiana* were used in medicinal baths to treat skin diseases, i.e., allergy and chicken pox. Li et al. [57] reported that medicinal baths are an important traditional method to cure and prevent common ailments among the traditional Yao communities of Jinping County, China. Medicinal baths are commonly used to prevent and treat skin diseases, rheumatic diseases, injuries, and gynecological disorders.

### Informant consensus factor

To determine the informant consensus factor (FIC), all the reported ailments were first grouped into 11 different disease categories on the basis of their use reports (Table 4). The uppermost FCI value is recorded for GIT diseases (0.41), followed by glandular diseases (0.34), dermatological disorder, and respiratory diseases (0.29). The mean FIC for all ailments categories was 0.17, which was similar to previously published studies reported from Pakistan [13, 58, 59]. Among the three major disease categories, GIT diseases were dominated with 154 use-reports, followed by dermatological disorders, and glandular complaints (120 and 103 use-reports, respectively) as mentioned in Table 4. Around 71.3% plant species were used to treat GIT disorders, followed by glandular complaints (65.9%), respiratory diseases (52.7%), ENEM diseases (40.3%), sexual diseases (31.0%), urinary problems, muscle and skeletal disorders (27.1% each), cardiovascular disorders (24%), body energizer (14%), and nervous disorders (7.8%). These results show that GIT and dermatological diseases are common in the study area. Similar findings have already been reported from other regions [31, 60]. Dermatological disorders with respect to FCI ranked as third category. The local people of the study area mostly prefer to use these plant-based treatments against skin diseases, insects bites, and scorpion sting.

**Table 4:** Informants consensus factor (FCI) by categories of ailments in the study area

Category of ailments	Nur.	% of use reports	Nt.	% of species	Nur-Nt	Nur-1	FCI
GIT diseases	154	23.2	92	71.3	62	153	0.41
Dermatological disorders	120	18.1	85	65.9	35	119	0.29
Glandular disorders	103	15.5	68	52.7	35	102	0.34
Respiratory diseases	73	11.0	52	40.3	21	72	0.29
ENEM diseases	43	6.5	40	31.0	3	42	0.07
Sexual diseases	42	6.3	35	27.1	7	41	0.17
Urinary disorders	36	5.4	35	27.1	1	35	0.03
Muscles and Skeletal disorders	32	4.8	28	21.7	4	31	0.13
Cardiovascular disorders	32	4.8	31	24.0	1	31	0.03
Body energizers	18	2.7	18	14.0	0	17	0.00
Nervous disorders	11	1.7	10	7.8	1	10	0.10
Mean FCI	–	–	–	–	–	–	0.17

### Relative frequency of citation and use report

In our study, relative frequency of citation (RFC) of the encountered plant species varied from 0.30 to 0.06 (Table 2). Maximum RFC value was calculated for species *W*. *somnifera* (0.30) followed by *Solanum surattense* (0.28), *Solanum nigrum* and *Azadirachta indica* (0.26 for each), *Ficus benghalensis*, *Morus nigra*, *M*. *alba* (0.23 for each), *Polygonum plebeium* (0.22), and *Tribulus terrestris* (0.19). *Melilotus indica* has the lowest RFC (0.06) in the area while Zahoor et al. [61] reported that *M*. *indica* has the highest RFC (0.78) which is contrary to our results. It can be seen that plants with the highest RFC are the most frequent medicinal plant in that region and majority of the people agreed by its medicinal value [58]. Use report value varied from 4 to 80 in the present study. *W*. *somnifera*, *S*. *surattense*, *S*. *nigrum*, *A*. *indica*, *M*. *alba*, *Ficus benghalensis*, *M*. *nigra*, *P*. *plebeium*, and *T*. *terrestris* were the most used plant species. Bibi et al. [58] reported the lowest use report of *S*. *nigrum* and *T*. *terrestris* (2 UR). The differences may be due to variation in vegetation and geo-climate of the area.

### Use value and potential of medicinal plants

The use value (UV) index is a method of the types of uses attributed to specific plant species and families for a population. In the present study, UV of the encountered plant species ranged from 0.84 to 0.1 (Table 2). The use value of *W*. *somnifera*, *S*. *surattense*, *S*. *nigrum*, *A*. *indica*, *M*. *nigra*, *F*. *benghalensis*, *P*. *plebeium*, and *M*. *alba* were 0.84, 0.82, 0.81, 0.74, 0.73, 0.73, and 0.71 respectively. Zahoor et al. [61] reported the lowest UV of *W*. *somnifera* (0.0085), *M*. *alba* (0.02), and *A*. *indica* (0.03), which is contrary to our results. The low UV of *Veronica polita****,*** *Malva parviflora*, *Cucumis melo*, and *B*. *diffusa* may be due to poor availability and lack of knowledge. These results were comparable with previous reports from Gujranwala and Hafizabad district, Pakistan [13, 15]. However, differences in most of the mentioned species and their quantitative values were also observed. In a field survey carried out by Ullah et al. [62], *Plantago ovata* and *Lawsonia inerm* were the most important species with the highest use value (0.98), while Bibi et al. [58] reported that *Berberis balochistanica* and *Citrullus colocynthis* had maximum use value (0.18 each), followed by *Descurainia sophia* (0.15). These differences may be due to variation in geo-climate, vegetation, traditional knowledge of informants, and their culture.

In Pakistan, majority of the people rely on medicinal plants to find treatments for their minor and major diseases [63]. Medicinal plants are growing abundantly in the wild, or some are cultivated on farmlands in the Punjab, Sindh, KPK, Baluchistan, and Azad Kashmir [64]. *W*. *somnifera* is an important wild medicinal plant used in Pakistan from the old time by the herbalists in making different medicines [65]. Withanolides extracted from *W*. *somnifera* are reported to be effective in protecting against β-amyloid-induced neurotoxicity [66]. In our study, leaves and berries of *S*. *nigrum* and *Solanum xanthocarpum* are commonly used for the treatment of gastric ulcers and cracked heel. Abbas et al. [67] assured the possible potential of antifungal as well as antimicrobial activity of fruit extracts of two Solanaceous plants (*S*. *nigrum* and *S*. *xanthocarpum*).

### Relative importance level

**Fig. 6:**
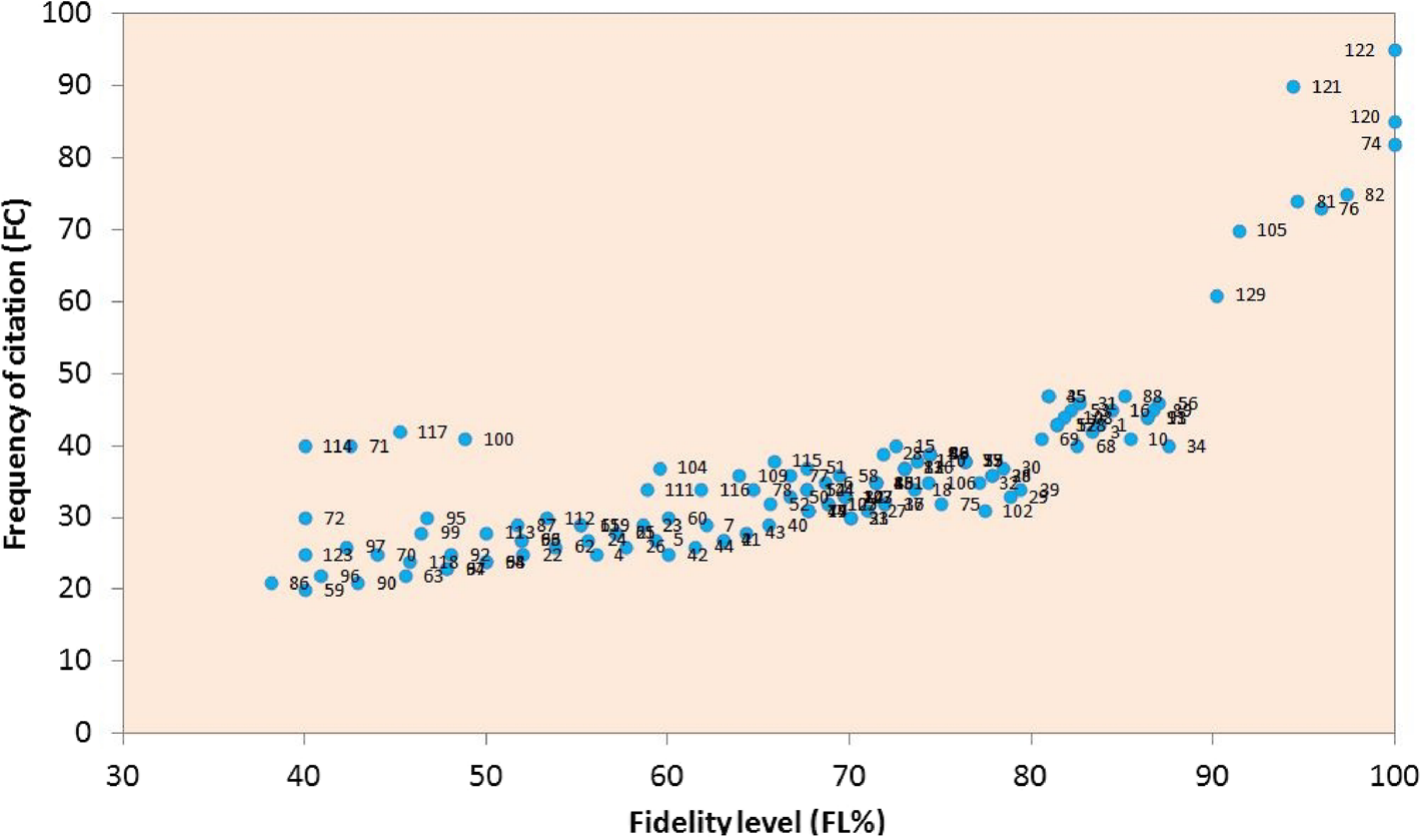
Relationship between numbers of informants and relative importance level (RIL). Numbers represent the plant names as they appear in Table 2

The importance of a plant species increases as it is used to treat more infirmities by the informants. For species mentioned by 20 to 48 respondents, the relative importance level (RIL) value increases directly with the increase in number of respondents. The RIL value of plant species mentioned by 48 or more respondents does not accelerate with the increased number of respondents (Fig. 6). One hundred twenty-three plant species, which were mentioned by 47 or less respondents, were classified as unimportant, whereas the 6 plant species cited by 48 respondents or more were declared as important. *W*. *somnifera*, *S*. *surattense*, *S*. *nigrum*, *A*. *indica*, *F*. *benghalensis*, *M*. *nigra*, *M*. *alba*, and *T*. *terrestris* were the most significant plant species with 1.0 RIL (Table 2). Umair et al. [13] reported the high popularity of *S*. *surattense*, *S. nigrum*, and *W*. *somnifera* in Hafizabad district, Pakistan. It can be seen that plants with high RIL value may attributed to their high efficacy and the awareness of local peoples which specifies their use as herbal medicine. These results were in agreement with previous reports on the medicinal use of plant species, e.g., among the local peoples of Negev district, Israel [26] and Palestinian area [28]. The high RIL value of plant species might be attributed to a wider geographic distribution, cultural knowledge and informant’s awareness.

### Fidelity level

**Fig. 7:**
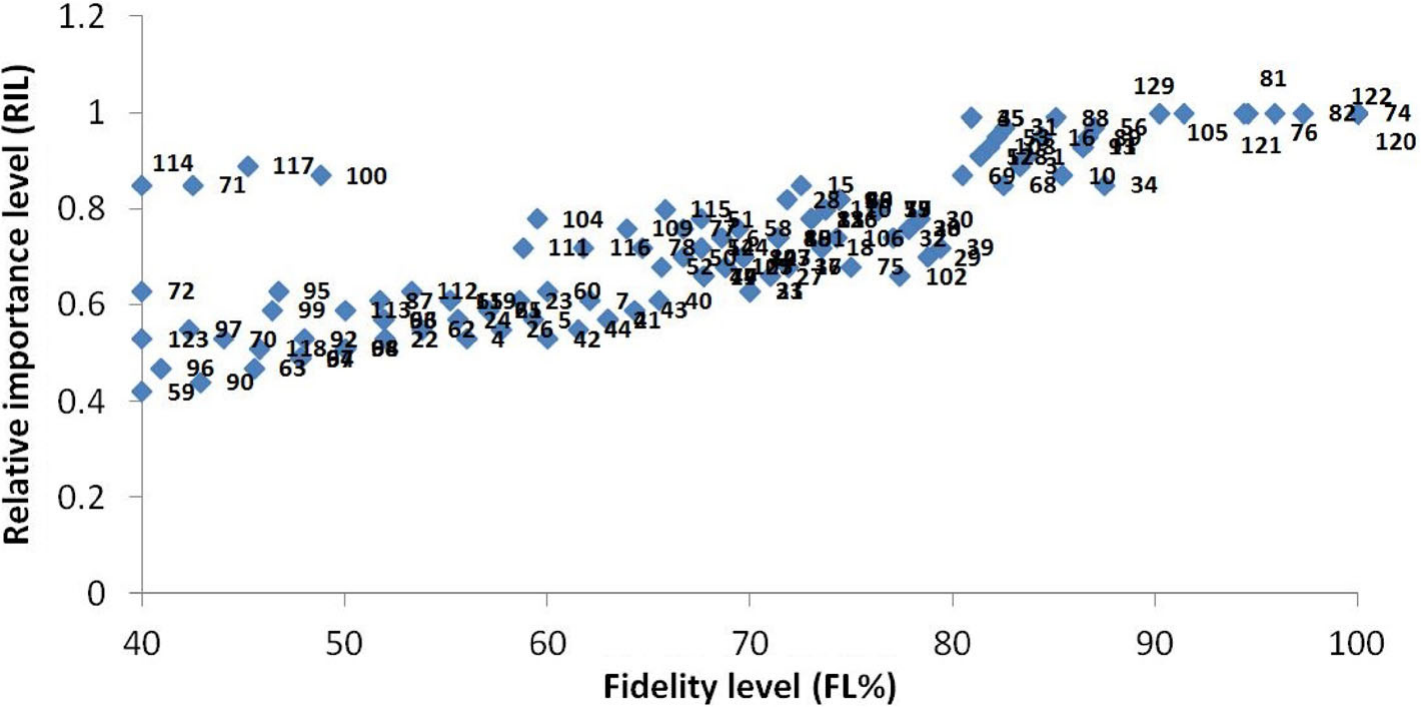
Relationship between numbers of informants claimed use of certain plant for particular disease. Numbers represent the plant names as they appear in Table 2

The fidelity level (FL) index is used to notify plant species that are most favored by the indigenous peoples to treat certain diseases [68]. Plant species with highest medicinal uses in a given area have maximum value of FL, i.e., 100%. In the present investigation, the FL value of the 129 plant species varied from 14.3 to 100% (Fig. 7). Generally, the high fidelity level of a species shows the abundance of a particular disease in a specific area and the utilization of plant species by the local people to treat it [58, 69]. The fidelity levels calculated for *M*. *nigra* (asthma), *F*. *benghalensis* (male sexual power), *M*. *alba* (cough), *S*. *surattense* (kidney stones), *P*. *plebeium* (pneumonia), and *T*. *terrestris* (urodynia) were 97.3, 95.9, 94.6, 94.4, 91.4, and 90.2%, respectively (Table 2). The most commonly used medical plants in the study area with 100% FL were *A*. *indica*, *S*. *nigrum*, and *W*. *somnifera*, which were used as blood purifier, to treat breast cancer and as stomachache, respectively. Comparatively, fidelity levels of these species were very high than previous reports [13] against gastrointestinal disorders, respiratory tract infections, urinary disorders, cardiovascular diseases, fever, pain, inflammation, and urological disorders with almost similar fidelity level. Additionally, in the present study, same species were reported to treat more diseases compared to previous report [14]. Plant species having high FL are seen as particularly interesting for biological, phytochemical, and pharmacological studies to evaluate and prove their validity to introduce novel drugs and herbal products.

### Corrected fidelity level

The corrected fidelity level (CFL) index is used to properly rank the plant species with different FL and RIL values. The resultant RIL values given in Table 2 were used as correction factor (CF) to adjust the FL values. The measured level of CFL of each plant species is mentioned in Table 2. The CFL value of only nine species was above 90. *W*. *somnifera*, *S*. *nigrum*, and *A*. *indica* were the highest utilized species with maximum CFL = 100, followed by *M*. *nigra*, *F*. *benghalensis*, *M*. *nigra*, *S*. *surattense*, *P*. *plebeium*, and *C*. *sativa* (97, 96, 95, 94, 91, and 90, respectively). This was probably due to increasing popularity of traditional medicines among the local peoples of the study area. Additionally, the respondents of the rural areas had more interaction and information about medicinal uses of plant species compared to urban areas. These findings were analogous to previous results from Hafizabad district [13], Negev district, Israel [26], and Palestinian area [28].

### Statistical analysis

The Pearson correlation coefficient (PCC) measures the power of a linear association between two component variables. The PCC index between UR and FC was 0.973 at *p* = 0.01 level. This reflects a highly significant positive association between the number of informants mentioning certain plant species and the number of applications reported. Furthermore, this shows that frequent use of plant species by the inhabitants tend to rise the applications number of usable species (*y* = 0.9269*x* − 13.637; correlation coefficient *r*^2^ = 0.947). In the present investigation, the value of *r*^2^ was 0.95 which indicates that around 95% of the variation in UR could be described in terms of the FC (Table 5). The plant species with higher FC value most have higher UR, such as *W*. *somnifera* and *S*. *surattense.* The present results are in accordance with previous reports. For example, Amjad et al. [43], Bano et al. [70], and Vijayakumar et al. [71] reported Pearson correlation coefficient between RFC and UV of 0.732, 0.638, and 0.881, respectively, with *r*^2^ = 0.54, 0.41, and 0.77 in respective order.

**Table 5:** Correlation coefficient between frequency of citation (FC) and use reports (UR)

Correlations		
Variables	UR	FC
UR		
Pearson Correlation	1	0.973^**^
Sig. (two-tailed)		0.000
*N*	129	129
FC		
Pearson Correlation	0.973^**^	1
Sig. (two-tailed)	0.000	
*N*	129	129

**Correlation is significant at the 0.01 level (two-tailed)

*r*^2^ = 0.947

### Novelty and future impact

To find the novelty index, data on ethnomedicinal uses of encountered species were compared with previous published reports from neighboring areas and Pakistan (Table 3). A total of 22 published studies were chosen for comparative analysis. *W*. *somnifera* shows maximum similarity with previously reported work from the surrounding areas [13, 15, 46, 61, 62, 72–78]. The ethnomedicinal data recorded from the study site discloses significant variations in the herbal preparation, dosage, applications, and utilization of plant parts recorded from other neighboring areas. About 12.47% uses of encountered species were comparable to previous reports. Moreover, 47% uses of the reported species were similar to previous study conducted in Hafizabad district [13]. Notably, 78.82% uses of the documented medicinal plant species were not reported in the previous studies used for comparative and novelty index obtained by dividing no use reports with all use reports for species multiply by 100. The percentage of novel uses (8.77%) of encountered species with respect to previous reports was obtained by dividing dissimilar use reports with all use reports for species multiply by 100. The comparison with neighboring areas depicted significant resemblances due to the traditional knowledge and culture exchange, while farther study areas had lower similarities due to the difference in traditions and cultures.

The comparative analysis between the uses of medicinal plants confirms the reported data.

To best of our knowledge, medicinal uses of *Polyalthia longifolia* (fever), *Pistia stratiote* (painful urination), *Schefflera arboricola* (blood circulation), *Ceratophyllum demersum* (diarrhea), *Najas graminea* (goiter and boils), *Vallisneria spiralis* (rheumatism), *Lemna minor* (antipyretic), *Marsilea minuta* (diarrhea), *Nelumbo nucifera* (ring worm), *Nymphaea lotus* (malarial fever), *Saccharum spontaneum* (skin eruption), *Ranunculus laetus* (antirheumatic), *Oligomeris linifolia* (throat pain and cough), *Misopates orontium* (tumors), and *Trapa bispinosa* (body energizer) were documented for the first time. Therefore, new medicinal uses of encountered species with high RIL and CFL value are suggested to be evaluated for in depth screening of bioactive compounds and related pharmacological activities.

## Conclusion

On the whole, 129 medicinal species used by the inhabitants of the investigation area to cure various diseases were reported. About nine plant species including *Withania somnifera*, *Solanum surattense*, *S*. *nigrum*, *Azadirachta indica*, *Ficus benghalensis*, *Morus nigra*, *M*. *alba*, *Polygonum plebeium*, and *Tribulus terrestris* were highly utilized with maximum UV, RFC, RIL, FL, and CFL values. A significantly positive correlation between UR and FC (*r* = 0.973 at *p =* 0.01) reflects strong association between the number of respondents mentioning a particular encountered species and uses reports. The determination value (*r*^2^) was 0.95, which indicates that 95% of variation in UR can be described in terms of the FC. Our findings revealed that the local people of the study area have close relation with their surrounding environment and still hold significant information on medicinal plant species. The comparative evaluation with published scientific reports exposed 10% resemblance and 14% dissimilarity to previous reported data; however, majority of the medicinal uses of the encountered plant species have rarely been reported before from this region. As metablomics and biomarker tools are increasingly used in drug discovery to understand the mechanism of disease pathology and improved the therapeutic strategies for upcoming challenges. Consequently, screening for biological active ingredients and in vivo*/*in vitro evaluation of pharmacological activities in reported medicinal plant species with high CFL and FL could be interesting for future drug discovery. Additionally, conservation measures should be taken to protect the flora of the River Chenab wetland, with special emphasis on medicinal plant species.

## Additional files

Additional file 1: Coordinates, area, population density and climate of the study sites. Source: Government of the Punjab [88]. (DOCX 17 kb)

Additional file 2: Ethnobotanical questionnaire form. (DOCX 17 kb)
