# Supplementary material for: Ethnomedicinal uses of the local flora in Chenab riverine area, Punjab province Pakistan
Source: J Ethnobiol Ethnomed. 2019 Feb 1;15:7. doi: 10.1186/s13002-019-0285-4 (PMC6359778; doi:10.1186/s13002-019-0285-4)
Supplement: Supplementary file 1 — Coordinates, area, population density and climate of the study sites. Source: Government of the Punjab [88]. (DOCX 17 kb) [file 13002_2019_285_MOESM1_ESM.docx]

**Additional file 1** Coordinates, area, population density and climate of the study sites

| **Study area** | **Coordinates** | **Elevation (ft)** | **Area (km^2^)** | **Population** | **Population density**  **(person per km^2^)** | **Average temperature (°C)** | **Average precipitation (mm)** |
| --- | --- | --- | --- | --- | --- | --- | --- |
| Hafizabad | 32° 7′ 12″ N  73° 40′ 48″ E | 800′ | 2,367 | 1,038,000 | 439 | 24.1 | 533 |
| Mandi-Bahuddin | 32° 58′ 82″ N,  73° 49′ 73″ E | 669′ | 2,673 | 1,160,552 | 421 | 23.9 | 576 |
| Gujranwala | 32° 15′ 44″ N,  74° 18′ 42″ E | 751' | 3,622 | 3,400,940 | 701 | 23.9 | 581 |
| Gujrat | 32° 57′ 11″ N,  74° 07′ 50″ E | 761' | 3,192 | 2,048,008 | 642 | 24 | 670 |
| Sargodha | 32° 8′ 37″ N    72° 67′ 19″ E | 626' | 5,854 | 2,665,979 | 460 | 23.8 | 410 |
| Sialkot | 32° 49′ 25″ N   74° 53′ 10″ E | 840' | 3,016 | 2,723,481 | 903 | 23.6 | 820 |

Source: [Government of the Punjab [88](#_ENREF_88)]
