# Supplementary material for: Ethnomedicinal uses of the local flora in Chenab riverine area, Punjab province Pakistan
Source: J Ethnobiol Ethnomed. 2019 Feb 1;15:7. doi: 10.1186/s13002-019-0285-4 (PMC6359778; doi:10.1186/s13002-019-0285-4)
Supplement: Supplementary file 2 — Ethnobotanical questionnaire form. (DOCX 17 kb) [file 13002_2019_285_MOESM2_ESM.docx]

**Additional file 2** Ethnobotanical questionnaire form

Date: __________

1. **Respondent information:**

Name of Respondents:_______________________

Age: ____________________________________

Address (Locality):_________________________

Gender: a) Male b) Female

Occupation: ______________________________

Qualification:_____________________________

Number of family members: _________________

Respondent category: a) Local informant b) Traditional health practitioners

Traditional health practitioners experience:

**B. Medicinal plant information:**

Local Name: _____________________________

Common Name: __________________________

Family: _________________________________

Life Habits/Life forms: _____________________

Therapeutic Use: Yes/No

Disease:_________________________________

Part of Plant Used:

a) Root b) Stem c) Leaves d) Fruit e) shoot f) whole plant

g) Bark h) Flower i) Rhizome j) Seed

Mode of utilization:_________________________

Application mode (dosage): ______________________________________________________________

_____________________________________________________________________________________
